# Caveolin‐3 deficiency associated with the dystrophy P104L mutation impairs skeletal muscle mitochondrial form and function

**DOI:** 10.1002/jcsm.12541

**Published:** 2020-02-23

**Authors:** Dinesh S. Shah, Raid B. Nisr, Clare Stretton, Gabriela Krasteva‐Christ, Harinder S. Hundal

**Affiliations:** ^1^ Division of Cell Signalling and Immunology, Sir James Black Centre, School of Life Sciences University of Dundee Dundee UK; ^2^ Institute of Anatomy and Cell Biology, School of Medicine Saarland University Homburg Germany

**Keywords:** Caveolin‐3, LGMD1C, Caveolinopathy, Mitochondria, Skeletal Muscle

## Abstract

**Background:**

Caveolin‐3 (Cav3) is the principal structural component of caveolae in skeletal muscle. Dominant pathogenic mutations in the Cav3 gene, such as the Limb Girdle Muscular Dystrophy‐1C (LGMD1C) P104L mutation, result in substantial loss of Cav3 and myopathic changes characterized by muscle weakness and wasting. We hypothesize such myopathy may also be associated with disturbances in mitochondrial biology. Herein, we report studies assessing the effects of Cav3 deficiency on mitochondrial form and function in skeletal muscle cells.

**Methods:**

L6 myoblasts were stably transfected with Cav3^P104L^ or expression of native Cav3 repressed by shRNA or CRISPR/Cas9 genome editing prior to performing fixed/live cell imaging of mitochondrial morphology, subcellular fractionation and immunoblotting, or analysis of real time mitochondrial respiration. Skeletal muscle from wild‐type and Cav3^−/−^ mice was processed for analysis of mitochondrial proteins by immunoblotting.

**Results:**

Caveolin‐3 was detected in mitochondrial‐enriched membranes isolated from mouse gastrocnemius muscle and L6 myoblasts. Expression of Cav3^P104L^ in L6 myoblasts led to its targeting to the Golgi and loss of native Cav3 (>95%), including that associated with mitochondrial membranes. Cav3^P104L^ reduced mitochondrial mass and induced fragmentation of the mitochondrial network that was associated with significant loss of proteins involved in mitochondrial biogenesis, respiration, morphology, and redox function [i.e. PGC1α, succinate dehyrdogenase (SDHA), ANT1, MFN2, OPA1, and MnSOD). Furthermore, Cav3^P104L^ myoblasts exhibited increased mitochondrial cholesterol and loss of cardiolipin. Consistent with these changes, Cav3^P104L^ expression reduced mitochondrial respiratory capacity and increased myocellular superoxide production. These morphological, biochemical, and functional mitochondrial changes were phenocopied in myoblasts in which Cav3 had been silenced/knocked‐out using shRNA or CRISPR. Reduced mitochondrial mass, PGC1α, SDHA, ANT1, and MnSOD were also demonstrable in Cav3^−/−^ mouse gastrocnemius. Strikingly, Cav3 re‐expression in Cav3KO myoblasts restored its mitochondrial association and facilitated reformation of a tubular mitochondrial network. Significantly, re‐expression also mitigated changes in mitochondrial superoxide, cholesterol, and cardiolipin content and recovered cellular respiratory capacity.

**Conclusions:**

Our results identify Cav3 as an important regulator of mitochondrial homeostasis and reveal that Cav3 deficiency in muscle cells associated with the Cav3^P104L^ mutation invokes major disturbances in mitochondrial respiration and energy status that may contribute to the pathology of LGMD1C.

## Introduction

Caveolae are inwardly‐directed flask‐like invaginations of the plasma membrane that are enriched in cholesterol and sphingolipids. Their structural integrity is critically dependent upon the presence of caveolin (Cav), an integral membrane protein that avidly binds cholesterol within the caveolar membrane domain.^1^, ^2^ Cav is present as three isoforms: Cav1 and Cav2, which are co‐expressed in most cell types and capable of forming hetero‐oligomers, and Cav3 whose expression is exclusive to skeletal, smooth, and cardiac muscle and capable of homo‐oligomerisation.^3^, ^4^ In addition to binding cholesterol, Cav isoforms also possess a scaffolding binding domain that allows them to bind and/or recruit functionally diverse molecules to caveolae where their modulation can affect numerous downstream signalling pathways and cellular responses.^5^, ^6^, ^7^, ^8^ Indeed, the importance of Cav1 and Cav3 in influencing diverse cell/tissue processes is highlighted by studies in *Cav1‐*deficient and *Cav3*‐deficient mice, which are not just devoid of cell surface caveolae but exhibit a range of phenotypes including muscle and pulmonary dysfunction and impaired lipid homeostasis.^9^ The significance of Cav3 in skeletal muscle physiology is further underscored by the numerous pathogenic mutations that have been identified in the *CAV3* gene that result in muscle caveolinopathies, including limb girdle muscular dystrophy type 1C (LGMD1C), rippling muscle disease, familial hypertrophic cardiomyopathy, distal myopathy and isolated hyperCKemia^10^. Significantly, dominant mutations in Cav3 are associated with reduced muscle Cav3 content that might have important implications for numerous Cav3‐dependent sarcolemmal processes and consequently upon disease severity.^10^

The single missense amino acid substitution from a proline to a leucine at residue 104 (Cav3^P104L^) is frequently associated with LGMD1C.^10^ Under normal circumstances, homo‐oligomers of wild‐type (WT) Cav3 would be routed to the sarcolemma/T‐tubule membrane *via* the ER‐Golgi pathway, but this targeting is notably defective for the Cav3^P104L^ mutant, which is retained within the Golgi network.^11^ Importantly, the formation of oligomers between WT and mutant Cav3 also impairs delivery of native Cav3 to the cell surface, which becomes targeted along with the mutated Cav3 protein for degradation by the ubiquitin‐proteasome pathway. In line with this, analyses of muscle from LGMD1C patients reveal a substantial decline (~95%) in Cav3 abundance,^12^ loss of cell surface caveolae, and marked disorganization of the T‐tubule system.^13^ These myopathic changes are likely to contribute to the atrophy and weakness of muscle seen in these patients and can be phenocopied in mice that are transgenic overexpressors for the Cav3^P104L^ mutant.^14^ While little is still known as to how the Cav3^P104L^ mutation might promote muscle atrophy. it has been reported that Cav3 represses myostatin signalling by blunting activation of its Type I cell surface receptor.^15^ Myostatin is a well‐known negative regulator of muscle mass,^16^ and this repression restrains the activity of a pathway that would otherwise promote muscle atrophy. This restraint is likely to be alleviated in muscle expressing the Cav3^P104L^ mutant owing to the substantial reduction in Cav3 that would result in a concomitant increase in myostatin signalling.^15^ Interestingly, very recent work has also linked expression of Cav3^P104L^ in C2C12 myotubes to reduced activation of Akt (a key insulin signalling intermediate^17^) and a diminution in glucose uptake and glycogen synthesis.^18^ These latter observations raise the possibility that disturbances in fuel and energy metabolism may also be contributing factors to the pathology of LGMD1C.

While expression of Cav proteins has been traditionally associated with cell surface caveolae, there is now considerable evidence supporting their localization within different subcellular compartments. Cav1, for example, is detectable in the Golgi,^19^ endosomes,^20^ cytosolic lipid droplets,^21^ secretory vesicles, and in close apposition to mitochondria in airway epithelial cells.^22^, ^23^ Cav1 is also an integral component of mitochondrial associated membranes in mouse liver where it has been implicated in the regulation of intracellular steroid and lipoprotein metabolism.^24^ More recently, Volonte *et al*. demonstrated that Cav1 is recruited to mitochondria during oxidative stress where it associates with a matrix‐oriented mitochondrial protease, AFG3L2, an interaction thought to form part of a stress‐response mechanism that may help maintain mitochondrial quality control.^25^ The notion that mitochondrial localized Cav may confer protection against cell stress and injury is also supported by studies in cardiomyocytes in which overexpression and increased localization of Cav3 to mitochondria are associated with enhanced calcium tolerance, improved respiratory function, and suppressed generation of reactive oxygen species (ROS).^26^, ^27^ Whether Cav3 similarly associates with mitochondria in skeletal muscle and whether loss of Cav3 impairs mitochondrial function is currently unknown. However, given that mitochondrial dysfunction has been linked to muscle atrophy during muscle inactivity,^28^ disturbances in mitochondrial biology may also be a feature contributing to the development and progression of muscle‐wasting conditions. In support of this proposition, there is growing evidence linking mitochondrial dysfunction to a number of muscular dystrophies, including Duchenne, spinal muscular atrophy, and the calpainopathy that induces limb girdle muscular dystrophy Type 2A (LGMD2A).^29^, ^30^, ^31^, ^32^ Whether it is also a trait of caveolinopathies such as LGMD1C has not yet been documented, but studies in cultured myoblasts and mice expressing the Cav3^P104L^ mutation have reported disordered glucose metabolism^18^ and altered expression of a number of mitochondrial proteins that regulate diverse aspects of mitochondrial structure and function.^11^ Precisely how these changes are linked to Cav3 dysfunction remain unclear, but we hypothesize that Cav3 is closely associated with mitochondria in skeletal muscle cells and that its dysfunction/loss, induced by the Cav3^P104L^ mutation, impairs mitochondrial form and function with important consequences for key energy‐dependent processes (e.g. protein synthesis) that may be significant in the pathology of LGMD1C. The studies described herein have tested this hypothesis.

## Materials and methods

### Chemicals and reagents

α‐Minimal essential medium (MEM), Dulbecco's modified Eagle's medium, OPTI‐MEM, Lipofectamine 2000, and antibiotic/antimycotic solution were from Invitrogen. Earle's Balanced Salt Solution was obtained from Sigma‐Aldrich. Foetal bovine serum was purchased from BioSera (France). Restriction enzymes and other DNA‐modifying enzymes were purchased from either New England Biolabs or Roche Diagnostics. Olignucleotides were purchased from the University of Dundee Oligo Synthesis Service (University of Dundee).

### Animals

As with other Cav3 knockout mouse strains that have been reported in the literature,^33^, ^34^ the mice used in the current study were generated by targeting exon‐2 of the Cav3 gene using the gene targeting approach detailed previously.^35^ Mice were held according to the German guidelines for the care and use of laboratory animals and experiments approved by the Saarland's institutional Animal Care and Use Committee. The phenotype of Cav3^−/−^ mice has been well documented,^9^ but adult mice exhibit myopathic changes resembling those described in patients with LCMD1C.^13^ These include muscle atrophy and changes in muscle ultrastructure (as highlighted by disorganization of the T‐tubule system, diminished muscle fibre size, and increased fibre necrosis) as well as reduced muscle function as judged by a significant decline in grip strength.^15^, ^33^ Despite muscle degeneration, Cav3^−/−^ mice have been shown to gain visceral fat with age contributing to an increase in body weight.^36^ In line with this latter report, the adult (15–20 weeks of age) male Cav3^*−/−*^ mice used in the present study were slightly heavier (34.7 + 0.9 g body weight, mean + SEM, *n* = 5) than age‐matched WT Cav3^*+/+*^ mice (29.1 + 0.2 g body weight, mean + SEM, *n* = 5). Mice were anaesthetized with an overdose of isoflurane, and the thoracic aorta was cut. Gastrocnemius muscle was excised from hind limbs and rapidly frozen in liquid N2 and stored at −80^°^C until required. For some experiments, C57BL/6 mice (15–20 week old) maintained at the University of Dundee in compliance with UK Home Office Animals (Scientific Procedures) Act 1986 were killed by CO_2_ inhalation and cervical dislocation and gastrocnemius muscle excised from hind limbs and processed immediately for histochemical staining or isolation of mitochondrial membranes.

### Muscle cell culture

L6 muscle cells were cultured as described previously^17^ in α‐minimal essential medium containing 2% (v/v) foetal bovine serum and 1% (v/v) antibiotic/antimycotic solution (100 units/mL penicillin, 100 μg/mL streptomycin, 250 ng/mL *amphotericin* B) at 37°C with 5% CO_2_. Consistent with previous studies,^37^, ^38^, ^39^ muscle cells expressing Cav3^P104L^ or in which Cav3 had been depleted using shRNA or CRISPR/Cas9 gene editing exhibit reduced fusion capacity. Consequently, comparative analyses of WT muscle cells and those in which Cav3 expression had been modified were conducted in myoblasts at Day 4 post‐seeding. For the purpose of the current studies, the term ‘muscle cells’ has been used interchangeably with myoblasts.

### Mitochondrial membrane isolation

Mitochondrial membranes and cytosolic fractions were prepared from mouse skeletal muscle as previously described.^40^ Briefly, gastrocnemius muscle was dissected and diced into small pieces (~50 mg) using scissors and subsequently homogenized using 20 strokes of an Eppendorf homogenizer. The homogenate was centrifuged at 700 *g* for 10 min at 4°C; the supernatant was transferred to a new tube and the pellet discarded. The supernatant was then centrifuged at 10 000 *g* for 20 min at 4°C, and the resulting supernatant (cytosolic fraction) was kept for further analysis. The mitochondrial‐enriched membrane pellet was washed twice with phosphate‐buffered saline (PBS) prior to being resuspended in lysis buffer. The mitochondrial and cytosolic fractions were then used for western blot analysis.

A mitochondrial‐enriched membrane fraction was obtained from L6 myoblasts using a mitochondrial isolation kit (#89874, Thermo Fisher Scientific) according to the manufacturer's instructions. Typically, cells were grown until confluence in 10 cm dishes. Muscle cells were harvested and subsequently homogenized using an Eppendorf homogenizer. The homogenized cell material was subject to centrifugation at 750 *g* for 10 min at 4°C. The resulting supernatant was subject to 14 000 *g* for 15 min at 4°C and stored at −20°C. The final pellet was an enriched mitochondrial fraction that was separated from the supernatant (cytosolic fraction). The mitochondrial pellet was solubilized in radioimmunoprecipitation assay buffer and stored at −20°C.

### Sodium dodecyl sulphate polyacrylamide gel electrophoresis and immunoblotting

Following appropriate experimental treatments, muscle cells were washed twice with ice‐cold PBS and then lysed as described previously.^17^ Protein concentrations in lysates and mitochondrial membrane preparations were determined using the Bradford method.^41^ Cell lysates and membrane preparations were subject to sodium dodecyl sulfate polyacrylamide gel electrophoresis (SDS‐PAGE) and separated proteins transferred onto PVDF membrane (Millipore) prior to incubation with the following primary antibodies at a dilution of 1:1000 except where indicated otherwise: actin (#A5060, 1:5000) was obtained from Sigma; ANT‐1 (#ab180715) and PGC1α (#ab54481) were from Abcam; MFN1 (#MMS‐5021) was purchased from Biolegend; Mitofusin 2 (MFN2) (#SC100560), succinate dehyrdogenase (SDHA) (#SC98253), and GAPDH (#SC32233, 1:5000) were purchased Santa Cruz; TOM20 (# 42406S), voltage‐dependent anion channel (VDAC) (#4661S), HA (#2367S), BiP (#C50B12), COX4 (#4580S), Catalase (#14097), Cytochrome C (#11940), RCAS1 (#12290), and manganase superoxide dismutase (MnSOD) (#D9V9C) were all purchased from Cell Signalling Technology; Cav1 (#610058), Cav3 (#610421), *DLP1*/Drp1 (#611112), and OPA1 (#612607) were from BD Biosciences. The α‐subunit of the Na,K‐ATPase antibody was from *Developmental Studies Hybridoma Bank* (University of Iowa). The puromycin antibody was from Kerafast (Boston, USA). Primary antibody detection was performed using appropriate horse‐radish peroxidase (HRP) conjugated secondary mouse (#7076S) or rabbit (#7074S) antibodies (1:5000) purchased from Cell Signalling Technology and visualised using enhanced chemiluminescence (Pierce‐Perbio Biotech, Tattenhall, UK) by exposure to autoradiographic film (Konica Minolta (Tokyo, Japan) or LICOR detection, respectively. Quantification of the immunoblot signals was carried out using Image J (NIH).

### Generation of stable caveolin‐3 silenced, caveolin‐3 knockout L6 myoblasts, and expression of HA‐GFP‐Cav3^P104L^, HA‐GFP‐CAV3, and mCherry‐IRES‐Cav3

Stable L6 Cav3 silenced cell lines were generated in a manner similar to that described previously^42^ using the pLKO.1‐puro vector and oligonucleotides indicated in *Table* 1. A non‐targeting control hairpin sequence was also included in these experiments (*Table* 1). Stable cell lines were established using early passage cells and, once established, were only used for a maximum of five passages.

**Table 1:** Oligonucleotide sequences

CRISPR	Antisense (5' to 3')	Sense (5' to 3')
Cav3 Knockout	CCGAAGAGCACACAGATCTG GAG	CATCAAGGACATTCACTGCA AGG
Oligos targeting the Cas9 nuclease to the Cav3 gene for double strand breaks		

Short Hairpin	Oligo 1 (5' to 3')	Oligo 2 (5' to 3')
Cav3 (shCav3)	**CCGGGGATCATCAAGGACATTCACT** CTCGAG **AGTGAATGTCCTTGATGATCCTTTTTG**	**AATTCAAAAAGGATCATCAAGGACATTCACT** CTCGAG **AGTGAATGTCCTTGATGATCC**
Control (shControl)	**CCGGCCTAAGGTTAAGTCGCCCTCG** CTCGAG **CGAGGGCGACTTAACCTTAGGTTTTTG**	**AATTCAAAAACCTAAGGTTAAGTCGCCCTCG** CTCGAG **CGAGGGCGACTTAACCTTAGG**
Oligos (5' to 3') are composed of sense and antisense sequences (boldface) with added overhangs to facilitate cloning into pLKO.1 puro, separated by a hairpin loop (underlined).		

NEBuilder HiFi DNA	Forward (5'to 3')	Reverse (5' to 3')
Fragment 1	acaagtccggactcgATGATGACCGAAGAGCACACAG	tagctcttaATGCAGAGCACC ACGGCC
Fragment 2	ctctgcatTAAGAGCTACCTG ATTGAG	cacattccacagggcTTAGTGATG GTGGTGATG
Overlapping primers incorporating the (P104L) mutation were designed for two Cav3 gene fragments that were then joined together and ligated into the vector using the NEBuilder HiFi kit.		

Gene Analysis	Forward Primer (5' to 3')	Reverse Primer (5' to 3')
COX4 (Mouse)	TTAACGAGAGCTTCGCCGAG	GAGGCCACATAAGACCCCAA
ND4 (Mouse)	AGCTCAATCTGCTTACGCCA	GCTGTGGATCCGTTCGTAGT
COX4	AATGTTGGCTACCAGGGCAC	GGGTAGTCACGCCGATCAAC
ND4	GAGGCAACCAAACAGAACGC	ATCATGTTGAGGGTAGGGGGT
Cardiolipin Synthase (CLS)	GATACCGAACTCTGCCAAC	GAAGCTGCCACCAAAATTAAC
Phosphatidylglycerol phosphate (PGP)	ACTCCTCCGACTCCTTATCC	ACCTCCACTGTGTCATCACC
PTPM1	GAAGGAATGGAAGAATGTGGG	GACTGGTACTTGAGAGCAAAC
CPD‐DAG Synthase	CTCAGTCACATCTTGTCATCC	AGAAGAAACCACCAATGAATCC
Tafazzin	CTCCTGAGTAGTGGAGGACA	TTCATATACTTGGTCCAGAAGCA

For the generation of the Cav3 IRES mCherry plasmid, the WT Cav3 gene was cloned into a pcDNA5D FRT/TO IRES mCherry vector [University of Dundee, MRC PPU Reagents and Services (DU50336)] using BamHI and XhoI restriction sites. This was transiently transfected into myoblasts using lipofectamine 3000 according to the manufacturer's instructions. Expression of the vector was initiated by incubation of cells with tetracycline at 1 μg/mL for 24 h.

Myoblasts lacking Cav3 were infected with retroviral particles containing  the pBABE hygro 3xHA GFP vector [University of Dundee, MRC PPU Reagents and Services (DU55543)] into which the Cav3 gene had been cloned using BamHI and NotI restriction sites. The pBABE hygro 3XHA construct was co‐transfected with GAG/POL and VSV‐G expression plasmids (Clontech, Saint‐Germain‐en‐Laye, France) into HEK 293T cells for the production of retroviral particles using Lipofectamine 2000 (Life Technologies) according to the manufacturer's instructions. The virus was harvested 48 h after transfection and applied to myoblasts in the presence of 10 μg/ml polybrene. Positive stable transformants were selected for using 500 μg/mL hygromycin and subsequently maintained in 50 μg/mL hygromycin thereafter.

For the generation of muscle cells expressing Cav3^P104L^, the proline 104 residue in the Cav3 gene within the pBABE hygro GFP vector was mutated to leucine using the NEBuilder HiFi DNA assembly kit as per the manufacturer's instructions using the oligonucleotides indicated in *Table* 1.

### Deletion of caveolin‐3 in L6 muscle cells using CRISPR/Cas9 Genome Editing

For cellular knockout of Cav3, specific sense and antisense guide RNA (gRNA) were designed (Table 1) to target exon1 of the *CAV3* gene (NC_005103.4) that were cloned into vectors supplied by the Division of Signal Transduction Therapy (University of Dundee). CRISPR/Cas9‐mediated deletion of Cav3 was accomplished by transfecting vectors containing the paired guides and Cas9 D10A into 10^6^ L6 myoblasts using polyethylenimine and selected for with 4 μg/mL puromycin over 48 h. After the initial selection process, a single colony was selected using FACS single cell sorting and Cav3 deletion in myoblasts confirmed by western blotting and immunofluorescence.

### Fixed and live cell imaging

For imaging of mouse skeletal muscle, gastrocnemius muscle was fixed in 4% (w/v) paraformaldehyde overnight, washed twice in PBS, and left in 30% (w/v) sucrose overnight. Muscle was covered in OCT mountant, frozen, and cryo‐sectioned (20 μm thickness) using a Leica cryostat. Muscle sections were incubated with Cav3 and TOM20 antibodies for 1 h and subsequently incubated with Alexa Fluor 488‐conjugated (#A‐11029) and Alexa Fluor conjugated 594 conjugated (#A‐11037) secondary antibodies (1:500) for 1 h and imaged using a Zeiss 710 Confocal Microscope.

#### For fixed cell imaging

Cells were grown to 70% confluence in 12‐well dishes on 13 mm coverslips and fixed in 4% (w/v) paraformaldehyde in PBS for 15 min at room temperature. Cells were permeabilized in PBS with 0.1% (v/v) Triton and blocked using 10% (v/v) goat serum in PBS for 60 min. Coverslips were then probed with Cav3 (1:300) and RCAS1 (1:400) antibodies for 1 h at room temperature in PBS containing 0.2% (w/v) bovine serum albumin and 0.02% (w/v) sodium azide. Coverslips were then washed three times and incubated with Hoechst (DNA stain 1:10 000) and secondary antibodies (1:500) conjugated to Alexa Fluor 488 or 594 for 1 h. Coverslips were finally washed three times with PBS and placed face down onto glass microscope slides with a drop of ProLong Diamond Antifade Mountant and sealed with nail varnish. Cells were imaged using a Zeiss 710 microscope.

#### For live cell imaging

Cells were seeded into μ‐Slide 8‐well chamber slides (ibidia, UK) and washed with fresh phenol red free MEM prior to incubation with 300 nM Mitospy Green FM (BioLegend, UK) or MitoTracker DeepRed FM (BioLegend, UK) for experiments with cells expressing GFP. Mitochondrial morphology was visualized in real time using a Zeiss 710 confocal microscope with a 60× oil‐immersion objective 37°C in a 5% CO_2_ chamber with excitation/emission set at 480 and 520 nm for Mitospy Green FM or 644 and 665 nm for Mitotracker Deep Red FM. For measurement of mitochondrial length, confocal images were processed using Volocity 6.3 Image Analysis Software (PerkinElmer). The software module can segment cellular components and give measurements such as mitochondrial number and classify them as condensed or elongated/tubular based on measurement of mitochondrial length. Mitochondria were categorized as either spheroid/fragmented in which mitochondria were equal to or less than 1 μm in length or tubular/elongated (including as part of a network), where mitochondrial length was greater than 1 μm. The number of mitochondria in each treatment condition was then determined and expressed as a percentage. To reduce any potential bias, a number of randomly chosen fields from multiple images were selected for quantification per experimental condition with each analysis being repeated in a minimum of three separate experiments or as indicated in the figure legends.

### Measurement of mitochondrial membrane potential, mitochondrial DNA, and citrate synthase activity

For measurement of mitochondrial membrane potential, muscle cells were seeded and grown in dark‐walled 96‐well dishes until confluent for 4 days. Cells were washed with PBS and in some experiments also treated with 5 μM FCCP (as a positive stimulus for collapsing the mitochondrial potential) prior to treatment with JC‐10 (20 μM) and incubated at 37°C, in 5% CO_2_ atmosphere for 45 min. The fluorescent intensities for both monomeric and J‐aggregates of JC‐10 were measured with excitation/emission set at 490/525 nm for monomer measurement and 540/595 nm for measurement of aggregates using a CLARIOstar plate reader prior to calculating the aggregate/monomer ratio.

For analysis/quantification of mitochondrial DNA, DNA was isolated from myoblasts using the DNeasy Blood and Tissue Kit as per the manufacturer's instructions. Equal amounts of DNA (100 ng) from each sample were then subject to quantitative polymerase chain reaction (qPCR) using the Syber Green method with primers designed to target genes encoded by nuclear DNA (COX4) and mitochondrial DNA (ND4). Data were expressed as a ratio of the ΔΔCt ND4 to the ΔΔCt of COX4. The forward and reverse primer sequences for the different gene targets are indicated in *Table* 1.

Citrate synthase (CS) activity was measured using a kit purchased from Sigma Aldrich, UK (MAK193). Muscle cells were treated as described above, and whole cell extracts were prepared at the end of the respective treatments. Thirty micrograms of cell extract protein was used for each enzymatic analysis (with measurements being conducted in triplicate for each experimental condition) at room temperature in 96‐well dishes. Enzyme activity was measured spectrophotometrically (at absorbance wavelength of 412 nm) using a μQUNT BIOTEK plate reader from LabTech. CS activity was calculated as per manufacturer's instructions.

### Analysis of reactive oxygen species

For mitochondrial ROS quantification, cells were seeded into dark‐walled 96‐well dishes and allowed to grow to confluence. Prior to analysis, cells were washed with PBS and then incubated with a fluorescent mitochondrial targeted superoxide probe (MitoSOX, 5 μM) at 37°C in a 5% CO_2_ atmosphere for 30 min. Upon binding superoxide, MitoSOX becomes fluorogenic emitting red fluorescence, which was quantified using a CLARIOstar plate reader at peak excitation/emission wavelengths of 510/580 nm and normalized to cellular protein.

### Mitochondrial respiration

Mitochondrial bioenergetics were measured in L6 myoblasts using a Seahorse XF24 analyser. WT L6 myoblasts or those that had been genetically manipulated (as indicated in the figure legends) were cultured on Seahorse culture plates and used to analyse basal respiration, adenosine triphosphate (ATP)‐Linked respiration, maximal respiratory capacity, and non‐mitochondrial respiration using modulators of cell respiration. Oligomycin (1 μM), FCCP (carobyl cyanide p‐trifluoromethoxyphenylhydrazone) (2 μM), rotenone (1 μM), and Antimycin (2 μM) were injected at the points indicated for ~6 min prior to measurement of oxygen consumption. The indicated mitochondrial parameters were normalized to cell protein.

### Analysis of cellular protein synthesis

Protein synthesis was measured as described by Goodman *et al*,^43^ by assaying puromycin incorporation into newly synthesized peptides. Briefly, myoblasts were pre‐incubated in the absence or presence of cycloheximide (50 μg/mL) prior to incubation with or without 1 μM puromycin for 15 min. At the end of this period, cells were lysed and lysates subjected to SDS‐PAGE and immunoblotting of PVDF membranes with a monoclonal antipuromycin antibody followed by incubation and detection with an antimouse HRP secondary antibody.

### Cholesterol Measurement

Cholesterol was quantified using the Thermo Amplex Red cholesterol assay kit according to the manufacturer's specifications. Twenty micrograms of whole cell lysate or 5 μg of mitochondrial‐enriched fractions were added to Amplex Red assay solution, which contains 300 μM Amplex Red reagent, 2 U/mL HRP as well as 2 U/mL cholesterol oxidase, 0.2 U/mL cholesterol esterase, 0.1 M of potassium phosphate, pH 7.4, 0.05 mM cholic acid, and 0.1% triton X‐100. After incubation of samples with an Amplex red assay solution for 30 min at 37°C protected from light, fluorescence was measured using a CLARIOstar plate reader set at 530/590 nm (excitation/emission). The relative concentrations of cholesterol were calculated using a calibration curve. Cholesterol abundance was presented as a fold change relative to control WT cells.

### Cardiolipin analysis

For analysis of cardiolipin, muscle cells were grown in dark‐walled 96‐well dishes or μ‐Slide 8‐well chamber slides until 70–80% confluent. Cells were subsequently stained with 100 nM 10‐N‐Nonyl Acridine Orange (NAO) for 30 min (protected from light) at 37°C with 5% CO_2_. At the end of this incubation period, cells were washed and the fluorescence intensity quantified using a CLARIOstar plate reader with excitation/emission set at 450/640 nm and normalized to the protein amount. Alternatively, muscle cells incubated with NAO were also incubated for the same period with Mitotracker DeepRed (to prevent overlap of fluorescence emissions with NAO). Cells were subsequently visualized using a Zeiss 710 confocal microscope at 37°C with 5% CO_2_, and 12‐bit images were obtained to attain higher pixel information allowing measurement and comparison of fluorescence intensity of NAO, normalized against Mitotracker Deep Red fluorescence intensity.

### Statistical analyses

Statistical analysis was performed using GraphPad Prism version 7 software using one‐way analysis of variance and Tukey post hoc test for multiple comparisons. Values were considered significant at *P* < 0.05.

## Results

### Caveolin‐3 is detectable within mitochondrial‐enriched membranes from mouse skeletal muscle and L6 myoblasts

**Figure 1:**
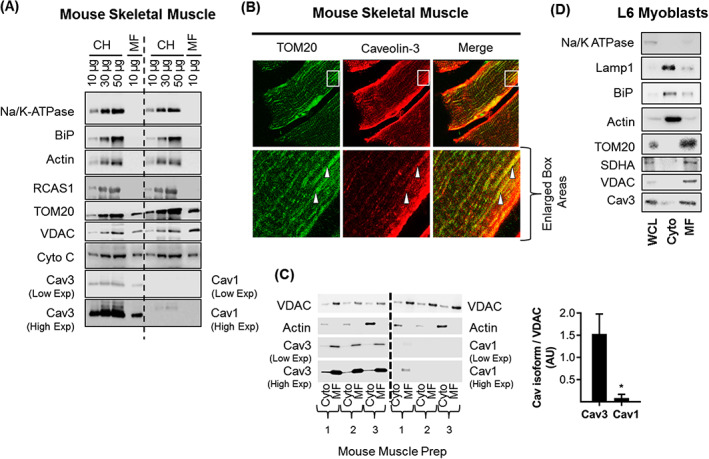
Cav3 is closely associated with mitochondria in mouse skeletal muscle and rat L6 myoblasts. Crude muscle homogenates (CH, 10, 30, 50 μg of protein) and isolated mitochondrial fractions (MF, 10 μg) from mouse gastrocnemius were used for sodium dodecyl sulfate polyacrylamide gel electrophoresis and immunoblotting using antibodies to the proteins indicated (A). Mouse gastrocnemius was sectioned and stained for mitochondrial marker TOM20 and Cav3. The field within the white box has been enlarged to highlight potential co‐localization (indicated by the white arrowheads) of TOM20 and Cav3 (B). Mitochondrial (MF, 10 μg protein) and cytosolic (Cyto, 10 μg protein) fractions isolated from gastrocnemius muscle of three separate mice were immunoblotted with antibodies to proteins indicated. Mitochondrial abundance of Cav1 and Cav3 were quantified (using the low exposure blots) relative to that of VDAC (C). Whole cell lysates (WCL, 5 μg protein), cytosolic (cyto, 5 μg protein) and mitochondrial fractions (MF, 5 μg protein) prepared from L6 myoblasts were subject to sodium dodecyl sulfate polyacrylamide gel electrophoresis and immunoblotted with antibodies to proteins indicated (D). The blots shown in (D) are representative of three separate experimental preparations.The graphical data represent mean ± SEM from a minimum of three separate experiments. Asterisks indicate a significant change (*P* < 0.05). Cav3, caveolin‐3; SDHA, succinate dehydrogenase subunit A; VDAC, voltage‐dependent anion channel.

To assess whether expression of Cav3 has any impact upon mitochondrial function in skeletal muscle cells, we initially investigated if it might be resident within mitochondrial‐enriched membrane fractions (MF) prepared from mouse gastrocnemius muscle and L6 myoblasts. Compared with a crude mouse muscle homogenate (CH), we observed immunoenrichment in the MF of VDAC, an outer mitochondrial membrane protein; TOM20, a key subunit of the TOM complex that is responsible for recognition and translocation of cytosolic synthesized mitochondrial preproteins; and Cytochrome C oxidase, a component of the mitochondrial respiratory chain (*Figure* 1A). It is important to stress that this comparative analysis of protein abundance in the CH and MF fractions is based on the relative signals obtained for equivalent protein loading (10 μg) on SDS‐gels for immunoblotting studies. Also noteworthy was our observation that actin (a cytosolic protein marker), the alpha subunit of the Na,K‐ATPase (a well‐established plasma membrane marker), RCAS1 (a Golgi marker^44^), and BiP [a major endoplasmic reticulum (ER) chaperone protein] were barely detectable in our isolated mitochondrial fractions indicating that these were largely free of membranes from other subcellular compartments. Strikingly, however, while we could detect an immunoreactive band for Cav3 in the CH, we also detected Cav3 in the MF fraction indicating that it is likely to be closely associated with membranes of mitochondrial origin. This latter proposition is further supported by immunohistochemical analysis, which showed that Cav3 and TOM20 costaining was detectable in muscle sections prepared from mouse gastrocnemius (*Figure* 1B). Although skeletal muscle also expresses Cav1, the abundance of this isoform was significantly less than that of Cav3 (*Figure* 1A). Consistent with this proposition, while Cav3 was detected in isolated MF from three separate mouse muscle preparations by immunoblotting, Cav1 immunoreactivity was difficult to detect even with prolonged exposure of blots. Ratiometric analysis of each Cav isoform relative to mitochondrial VDAC showed that Cav3 abundance in the MF was ~18‐fold greater than Cav1 (*Figure* 1C). Both Cav isoforms and VDAC were largely absent from an actin‐enriched cytosolic fraction from mouse gastrocnemius (*Figure* 1C).

To further substantiate the observed mitochondrial‐Cav3 association that we see in mouse muscle, we utilised a non‐mechanical, reagent‐based method that allows isolation of mitochondria from rat L6 myoblasts. The MF isolated by this procedure is, by comparison with a whole cell lysate (WCL) or cytosolic (cyto) fraction, highly enriched in VDAC, SDHA, and TOM20, but depleted of cytosolic (actin), plasma membrane (Na,K‐ATPase), ER (BiP), and lysosomal (Lamp1) protein markers. Furthermore, while we could detect Cav3 in the WCL, we also observed that its abundance was enriched within the MF from L6 cells (*Figure* 1D). Consistent with this observation, in separate experiments utilising L6 myoblasts overexpressing GFP‐tagged Cav3, we could visualize costaining of GFP‐Cav3 with membranes structures that had been stained with Mitotracker Red, a fluorescent dye that specifically labels up mitochondria within live cells (Supporting Information, *Figure* *S1*).

### Cav3P104L localizes to the Golgi in L6 muscle cells and affects mitochondrial morphology

**Figure 2:**
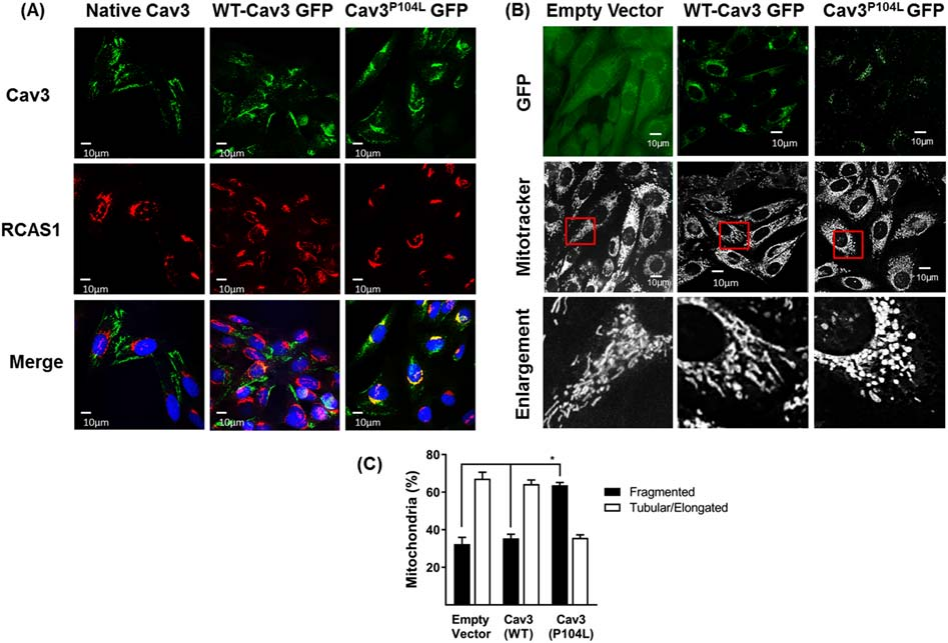
Expression of Limb Girdle Muscular Dystrophy‐1C Cav3 mutation (P104L) localizes Cav3 to the Golgi and causes mitochondrial fragmentation. L6 myoblasts expressing WT‐Cav3‐GFP or Cav3^P104L^‐GFP were fixed and immunostained for RCAS1 (red, a Golgi marker) and stained with DAPI (blue, nuclear stain) while wild‐type (WT) cells not expressing GFP were immunostained using antibodies to Cav3 (green), and visualized using confocal microscopy, on three separate experiments with a minimum of 6 images per sample (A). L6 myoblasts stably expressing an empty GFP vector, WT‐Cav3‐GFP or Cav3^P104L^ ‐GFP were stained with Mitotracker Deep Red and visualized using live cell confocal microscopy. Enlarged images (derived from the fields within the indicated red boxes) highlight changes in mitochondrial morphology (B). Mitochondrial length was quantified using Volocity software and presented as elongated/tubular if greater than 1 μm and fragmented if less than 1 μm in length. Data are presented as mean ± SEM from a minimum of three separate experiments with analyses of 15–20 randomly chosen visual fields for each condition in each experiment (C). Asterisks indicate a relative significant change (*P* < 0.05) between the indicated bars. Cav3, caveolin‐3; GFP, green fluorescent protein.

To assess how expression of the Cav3^P104L^ mutation might impact upon mitochondrial biology, we expressed GFP‐tagged versions of WT Cav3 and the Cav3^P104L^ mutant in L6 myoblasts and explored how their localization compared with that of endogenously expressed Cav3. *Figure* 2A shows that native Cav3 localizes to the peripheral boundary of myoblasts, which most likely reflects plasma membrane labelling but was also detectable within myoblasts. A very similar distribution was observed for ectopically expressed WT‐Cav3‐GFP indicating that the GFP tag does not interfere with the cellular targeting/localisation of Cav3. By comparison, while surface and intracellular labelling of the GFP‐tagged Cav3^P104L^ mutant was very modest, it was predominantly perinuclear in localization. Previous studies have suggested that the P104L mutation leads to retention of unstable Cav3 aggregates within the Golgi complex.^45^ Consistent with this observation, co‐immunostaining of myoblasts for RCAS1 showed that this Golgi protein strongly colocalized with the Cav3^P104L^ mutant, but not with native or ectopically expressed GFP‐tagged WT‐Cav3 proteins (*Figure* 2A).

Because native Cav3 was detectable within membranes of mitochondrial origin and we find that the Cav3^P104L^ mutant congregates within the Golgi, we subsequently assessed its impact on mitochondrial morphology. For this, we performed live cell imaging to visualize mitochondria using Mitotracker DeepRed, which is excluded from nuclei but accumulates within mitochondria. Myoblasts expressing the empty vector or that encoding the GFP‐tagged WT‐Cav3 exhibited mitotracker staining in which the mitochondria appeared predominantly (~65%) tubular/elongated (including being part of a network) and greater than 1 μm in length. By contrast, myoblasts expressing Cav3^P104L^ show a striking shift in mitochondrial morphology to one that was largely fragmented/spheroid (>60%) in which mitochondria were equal to or less than 1 μm in length (*Figure* 2B and 2C).

### Effects of expressing CavP104L on mitochondrial function

**Figure 3:**
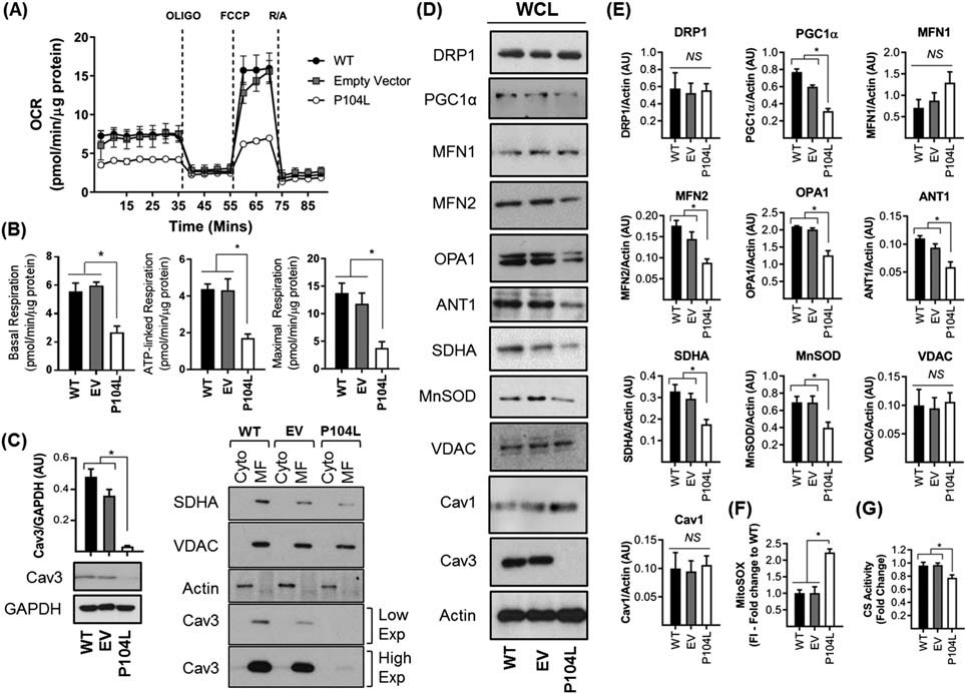
Effects of expressing the Cav3^P104L^ mutation on mitochondrial respiration and protein expression in L6 muscle cells. Wild‐type L6 muscle cells or those stably expressing an empty vector (EV) or one encoding Cav3^P104L^ were used for analysis of cell respiration using Seahorse technology. The oxygen consumption rate (OCR) trace shown in (A) is representative from a single experiment in which each point represents mean ± SEM of a triplicate measurement. Oligomycin (1 μM), FCCP (1 μM), and a mixture of Rotenone (1 μM)/Antimycin (2 μM) were added for determination of basal, ATP‐linked, and maximal respiration (B). The abundance of Cav3 and GAPDH (used as loading control) in WT, EV, and Cav3^P104L^ expressing L6 muscle cells was determined by immunoblotting. The bar graph quantifies Cav3 abundance relative to GAPDH (mean ± SEM) (C). Cytosolic (Cyto, 5 μg protein) and mitochondrial‐enriched fractions (MF, 5 μg protein) from WT, EV, and Cav3^P104L^ expressing myoblasts were immunoblotted with antibodies directed to the proteins indicated. The Cav3 blots show two separate image exposures. Whole cell lysates (WCL, 30 μg protein) from WT, EV, and Cav3^P104L^ expressing myoblasts were subject to sodium dodecyl sulfate polyacrylamide gel electrophoresis and immunoblotting with antibodies to proteins indicated (D) and their abundance quantified from a minimum of three separate experiments relative to actin (gel loading control) using Image J software (E). Analysis of superoxide content (F) was determined using fluorescence intensity (FI) of MitoSOX and citrate synthase (CS) activity in myoblasts was used as a proxy for mitochondrial mass (G). All graphical data represent mean ± SEM from a minimum of three separate experiments. Asterisks indicate a significant change (*P* < 0.05), whereas the NS notation signifies no significant change. Cav3, caveolin‐3; SDHA, succinate dehydrogenase subunit A; MnSOD, manganase superoxide dismutase; WT, wild type; VDAC, voltage‐dependent anion channel.

To determine whether the observed shift in mitochondrial morphology is accompanied by changes in mitochondrial function, we studied cell respiration in myoblasts in real time using a Seahorse extracellular flux analyser. This approach measures oxygen consumption rates (OCR) before and after addition of compounds that target Complex I and III of the respiratory chain, the ATP synthase, or FCCP [which uncouples mitochondrial oxidative‐phosphorylation (OXPHOS)] to allow analysis of numerous mitochondrial parameters. Myoblasts expressing WT‐Cav3 or those expressing the empty vector (EV) exhibit very similar OCR traces, whereas those expressing Cav3^P104L^ display a significant reduction in basal, ATP‐linked, and maximal respiration (*Figure* 3A and 3B). It is noteworthy that expression of Cav3^P104L^ results in a substantial reduction in cellular Cav3 as determined by immunoblotting of whole cell lysates (*Figure* 3C), which is most likely a result of aggregation within the Golgi and subsequent degradation by the proteasome. In line with the Cav3 loss seen in cells expressing Cav3^P104L^, we also observe a substantial decline in Cav3 in the mitochondrial‐enriched fraction prepared from these cells (*Figure* 3C).

To understand what might contribute to the change in morphology and the decline in mitochondrial respiratory capacity in Cav3^P104L^ expressing myoblasts, we assessed the expression of a number of proteins relevant to mitochondrial biology. While we could not detect any significant differences in VDAC, Drp1, or Mitofusin 1 abundance, we saw significant loss of MFN2 and OPA1. With the exception of VDAC, the latter proteins are involved in regulating mitochondrial dynamics. Drp1 facilitates mitochondrial fission, whereas mitofusins and OPA1 are important regulators of mitochondrial clustering and fusion.^46^ The reduction in MFN2 and OPA1 expression is consistent with a shift in mitochondrial dynamics favouring increased fission and is in line with the greater mitochondrial fragmentation seen in Cav3^P104L^ expressing myoblasts (*Figure* 2B and 2C). We also observed a significant decline in the abundance of the adenine nucleotide translocator [ANT1, which exchanges mitochondrial synthesized ATP for cytosolic adenosine diphosphate (ADP)], SDHA, and MnSOD, which represents a key mitochondrial antioxidant enzyme. Reduction of the latter would be anticipated to impair the capacity of cells to detoxify mitochondrial generated superoxide, which was significantly increased (>two‐fold) in Cav3^P104L^ expressing myoblasts (*Figure* 3F). Our analysis also revealed a reduction in the expression of PGC1α, a key transcriptional regulator of mitochondrial biogenesis that may contribute to a decline in mitochondrial respiratory capacity via a reduction in mitochondrial density.^47^ To explore this possibility, we assayed the activity of CS, an enzymatic marker of mitochondrial mass. Cav3^P104L^ myoblasts exhibit a modest, but significant decline in CS activity (Figure *3* *G*).

The data presented in *Figures* 2 and 3 indicate that expression of Cav3^P104L^ causes a substantial loss of native Cav3 and that this is associated with significant disturbances in mitochondrial morphology, respiratory function, and expression of key mitochondrial proteins. To further substantiate the idea that loss of Cav3 is what is likely to drive mitochondrial dysfunction, we performed two independent but complementary genetic approaches to stably deplete (using shRNA) or knockout (KO, using CRISPR/Cas9 editing) Cav3 in L6 muscle cells. The data presented in Supporting Information, *Figures* *S2*, S3 and *S4* show that irrespective of which genetic approach was used to reduce Cav3 in L6 myoblasts, we observe very similar disturbances in mitochondrial biology to that seen in Cav3^P104L^ expressing myoblasts (*Figures* 2 and 3). Furthermore, analysis of the aggregate to monomer distribution ratio of JC10, a potentiometric fluorescent dye that accumulates within mitochondria, revealed that compared with WT cells, this was significantly reduced in shCav3 and Cav3KO myoblasts (Supporting Information, *Figure* *S2* *D*). This latter observation implies that Cav3 deficiency is associated with a marked depolarisation of the mitochondrial membrane potential. A corollary of the observed reduction in proteins that support mitochondrial biogenesis, morphology, respiratory function, and adenine nucleotide exchange is that myoblasts lacking Cav3 are also likely to exhibit an energy deficit. Consistent with this proposition, the ATP:ADP ratio was significantly lower in both shCav3 and Cav3KO myoblasts (Supporting Information, *Figure* S2E).

### Analysis of caveolin‐3‐knockout mouse muscle

**Figure 4:**
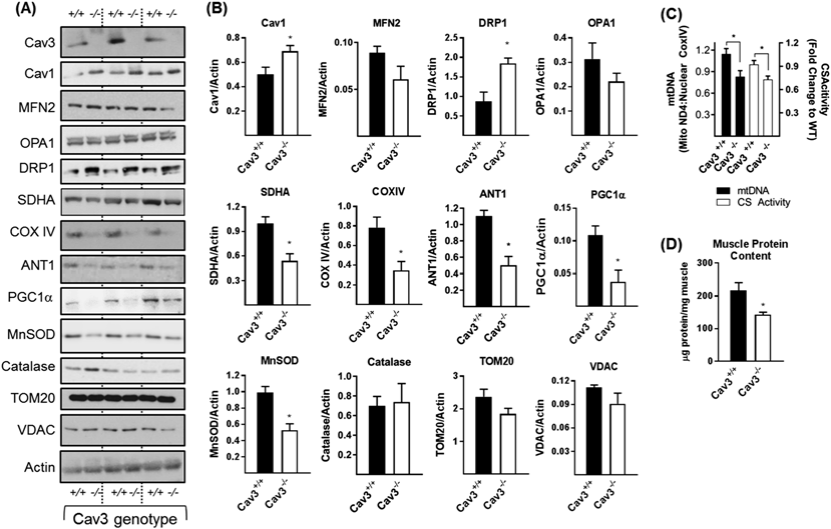
Effects of Cav3 deficiency upon proteins linked to mitochondrial function and upon mitochondrial mass in mouse skeletal muscle. Crude muscle homogenates prepared from gastrocnemius muscle of three wild type (WT, +/+) and three Cav3 knockout (Cav3KO, −/−) mice were subject to sodium dodecyl sulfate polyacrylamide gel electrophoresis and immunoblotted with antibodies to proteins shown (A) and protein abundance quantified relative to actin (used as a gel loading control) using Image J software (B). The data in (B) (mean ± SEM) is based on analysis of five WT and five Cav3KO mice. Alternatively, muscle tissue from mice was used for analysis of mitochondrial DNA or citrate synthase activity (C) or total muscle protein (D) as described in the methods section. Values in (C and D) are expressed as mean ± SEM from a minimum of three mice. Asterisks indicate a significant change (*P* < 0.05) to the appropriate WT data or between the indicated bars. Cav3, caveolin‐3; SDHA, succinate dehydrogenase subunit A; MnSOD, manganase superoxide dismutase; WT, wild type; VDAC, voltage‐dependent anion channel.

Our cell‐based studies show that Cav3 loss is associated with disturbances in mitochondrial morphology and function that may be driven, in part, by changes in the expression of key mitochondrial proteins. To assess whether comparable changes in protein expression are evident *in vivo,* we immunoblotted crude homogenates prepared from gastrocnemius muscle of WT^(+/+)^ and Cav3KO^(−/−)^ mice. Unlike myoblasts, loss of Cav3 in mouse muscle induced a modest, but significant, compensated increase in Cav1 (*Figure* 4A and 4B). Furthermore, while we saw no significant changes in the expression of MFN2 or OPA1 between muscle from Cav3^(+/+)^ and Cav3^(−/−)^ mice, there was a robust increase in the abundance of Drp1, a profission protein and notable loss of SDHA and COX IV (two key respiratory proteins) and that of MnSOD, ANT1 and PGC1α (*Figure* 4A and 4B). Consistent with our cell‐based data (Supporting Information, *Figure* *S2*), the expression of TOM20 and VDAC was unaffected by loss of Cav3 in mouse muscle (*Figure* 4A and 4B).

The reduced expression of respiratory proteins in muscle of Cav3KO mice may suggest a decline in respiratory drive/mitochondrial mass. While we did not validate whether mitochondrial respiration was impaired in intact mitochondria isolated from skeletal muscle of Cav3^−/−^ mice, a diminished mitochondrial function/mass will have implications for energy‐demanding processes such as protein synthesis whose reduction may contribute to the degeneration and weakness of muscle reported in these mice.^15^, ^33^, ^34^ Consistent with this proposition, analysis of mitochondrial density (as judged by measurement of mitochondrial DNA and CS activity) and total muscle protein expressed per milligram of muscle weight revealed these were significantly reduced by ~22% and 35%, respectively, in gastrocnemius muscle of Cav3^−/−^ mice (*Figure* 4C and 4D).

### Effects of caveolin‐3 deficiency on cellular protein synthesis

**Figure 5:**
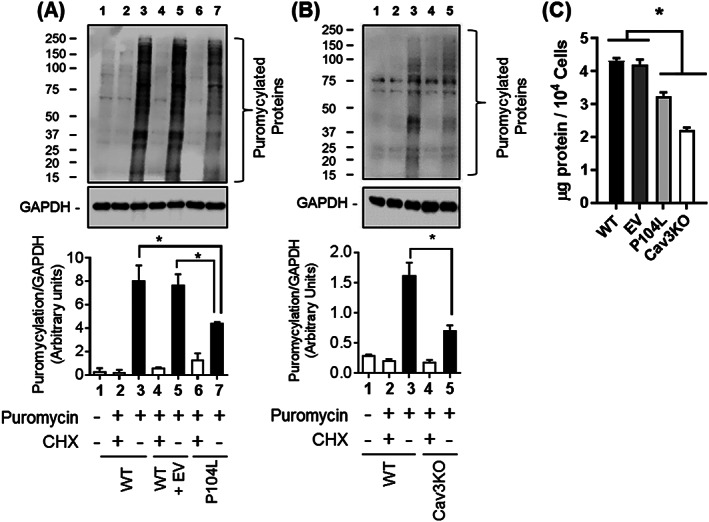
Cav3 depleted cells exhibit reduced protein synthetic capacity. For analysis of protein synthesis, wild‐type L6 myoblasts stably expressing an empty vector (EV) or one encoding Cav3 P104L or myoblasts in which Cav3 was deleted using CRISPR/Cas9 (Cav3KO) were incubated in the absence or presence of puromycin for 15 min (Lanes 2–7) or subject to pre‐treatment with cycloheximide for 15 min prior to puromycin exposure [Lanes 2, 4, and 6 in (A) and Lanes 2 and 4 in (B)]. Following treatment, myoblasts were lysed and lysates (30 μg protein) from (A) WT, WT + EV, Cav3 P104L expressing or (B) Cav3KO myoblasts were subject to sodium dodecyl sulfate polyacrylamide gel electrophoresis and immunoblotting with antibodies to puromycin and GAPDH and their abundance quantified from three separate experiments relative to GAPDH (a gel loading control) using Image J software. For total protein content 10^4^ myoblasts (WT, EV, P104L, and Cav3KO) were counted and protein assessed using the Bradford method and expressed as μg/10^4^ cells (C). All bar graph data represent mean ± SEM from three separate experiments. Asterisks indicate a significant change (*P* < 0.05). CHX, cycloheximide; EV, empty vector; WT, wild type.

To assess whether the reduced mitochondrial density and energy balance seen in Cav3^P104L^ and Cav3KO myoblasts compromises the activity of energy‐demanding processes, we assessed cellular protein synthesis using the non‐isotopic SUnSET technique,^43^ which assays incorporation of puromycin into newly synthesized polypeptides. *Figure* 5A and 5B show that the abundance of puromycylated proteins in Cav3^P104L^ expressing myoblasts or that in Cav3KO myoblasts was notably lower compared with that seen in WT L6 myoblasts. As expected, prior treatment of L6 myoblasts with cycloheximide inhibits protein synthesis as judged by the striking reduction in puromycylation (compare Lanes 2 and 3 in *Figure* 5A and 5B). In line with the lower protein synthesis, total protein content in Cav3^P104L^ expressing myoblasts or that in Cav3KO myoblasts was significantly less compared with of WT myoblasts when assessed using equivalent myoblast numbers for the protein assay (*Figure* 5C).

### Cellular re‐expression of caveolin‐3 in a caveolin‐3 null background rescues the loss in mitochondrial morphology and respiratory function

**Figure 6:**
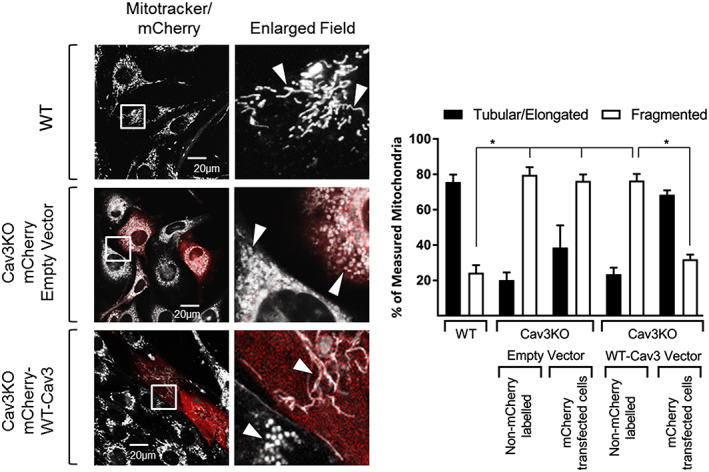
Effects of Cav3 re‐expression on mitochondrial morphology in L6 muscle cells. Myoblasts in which Cav3 had been deleted by CRISPR/Cas9 (Cav3KO) were transiently transfected with either a control mCherry empty vector or a mCherry vector encoding wild‐type Cav3 prior to staining with Mitotracker Green and imaging by confocal microscopy and comparative analysis with Mitotracker stained wild type myoblasts (WT). Images depict mitochondrial morphology in L6 myoblasts from WT and Cav3KO cells. Myoblasts that were transiently transfected and expressing the mCherry appear red. The fields contained within the white boxes have been are enlarged to highlight differences in morphology. White arrow heads draw attention to changes mitochondrial morphology of interest. Mitochondrial length in WT cells and Cav3KO cells in which the empty vector or that containing the WT‐Cav3 insert was measured using Volocity software and judged as tubular/elongated if greater than 1 μm in length and fragmented if less than 1 μm. In addition, cells within the same assay that had not taken up the mCherry vector were also used for comparative measurement of mitochondrial length and compared against WT and transfected cells. Data in the bar chart are presented as mean ± SEM from three separate experiments in which at least 12 randomly chosen visual fields were analysed. Asterisks indicate a significant change (*P* < 0.05) between the indicated bars.

Given that Cav3 loss is associated with increased fragmentation of the mitochondrial network and reduced mitochondrial respiratory function, we subsequently assessed if this could be mitigated by ectopic re‐expression of WT Cav3 into a Cav3 null background. For these studies, we transiently transfected mCherry‐WT‐Cav3 (or the empty mCherry vector) into myoblasts in which Cav3 had been deleted by CRISPR/Cas9 prior to mitochondrial staining and live cell imaging. Mitotracker labelling highlighted a predominantly (~75%) elongated and tubular mitochondrial network in WT L6 myoblasts. By contrast, the mitochondrial network was largely fragmented in Cav3KO cells irrespective of whether they had been transfected with the empty mCherry vector (red labelled myoblasts) or not (non‐labelled cells). Strikingly, however, Cav3KO myoblasts that had been successfully transfected with the mCherry‐WT‐Cav3 (i.e. those staining red) exhibit mitochondria that were part of an elongated/tubular network that had some semblance of that observed in WT cells. Importantly, neighbouring myoblasts that had not been transfected/labelled with the mCherry‐WT‐Cav3 vector still retained the highly fragmented mitochondrial morphology (*Figure* 6A and 6B).

**Figure 7:**
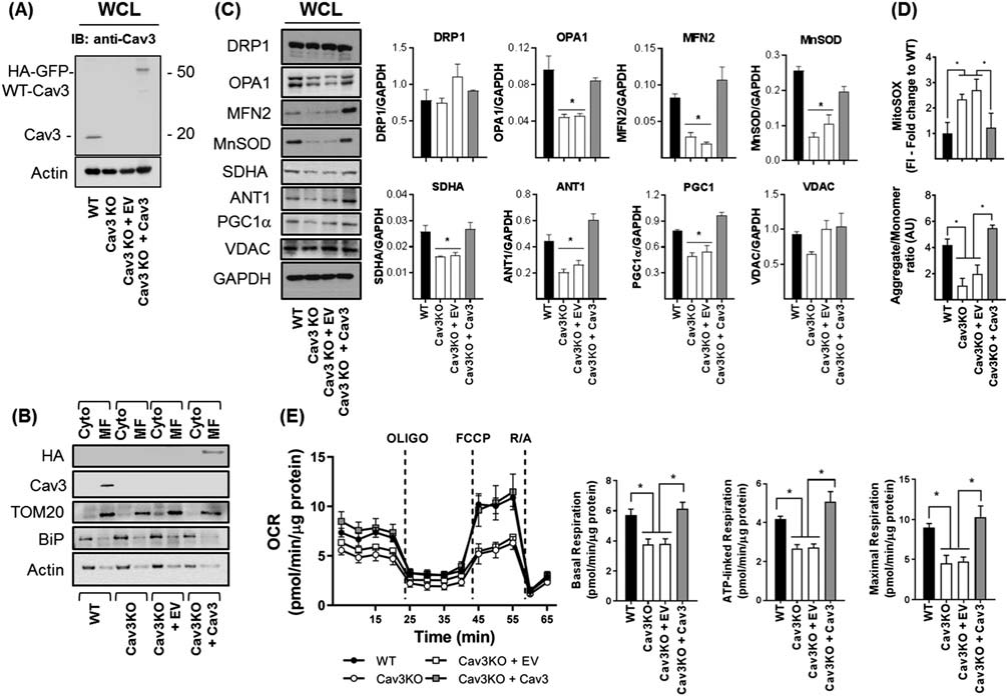
Effects of Cav3 re‐expression on mitochondrial protein expression, membrane potential, superoxide and mitochondrial respiration. Myoblasts in which Cav3 had been deleted by CRISPR/Cas9 (Cav3KO) were infected with retroviral particles containing an empty vector (Cav3KO + EV) or a vector containing HA‐GFP tagged WT‐Cav3 (Cav3KO + Cav3) and stable transformants isolated by antibiotic selection prior to: (A) immunoblotting whole cell lysates (WCL) with antibodies against Cav3 or actin (used as a gel loading control) antibodies; (B) isolation of cytosolic (Cyto) and mitochondrial membranes and immunoblotting using antibodies to proteins indicated; (C) lysis and immunoblotting of WCL with antibodies to proteins shown and quantification of their abundance relative to GAPDH (used as a gel loading control) using Image J software; (D) determination of superoxide content using fluorescence intensity (FI) of MitoSOX and mitochondrial membrane potential monitored by  spectral analysis of JC‐10 aggregate:monomer content from three separate  experiments (for this experiment GFP would interfere with the JC‐10 probe and therefore wild type Cav3 was cloned into pcDNA3.1 using HindIII and XhoI Restriction sites and an empty vector or a vector containing Cav3 were transiently transfected in L6 muscle cells and selected for using G418 before JC‐10 probe incubation); and  (E) analysis of mitochondrial respiration to assess basal oxygen consumption rate using Seahorse technology. Oligomycin (1 μM), FCCP (1 μM) and a mixture of Rotenone (1 μM)/Antimycin (2 μM) were added at the times indicated by dotted lines to determine basal, ATP‐linked and maximal respiration. The Seahorse trace shown in (E) is from a single experiment in which each point represents the mean ± SEM from triplicate analyses. All bar graph data in (E) represent the analysis of three individual experiments (values are mean ± SEM). Asterisks indicate significant change (*P* < 0.05) to the WT bar value or between indicated bars. Cav3, caveolin‐3; EV, empty vector; SDHA, succinate dehydrogenase subunit A; WT, wild type; VDAC, voltage‐dependent anion channel.

To determine whether expression of key mitochondrial proteins and respiratory function could be rescued by re‐expression of Cav3, we generated a stable Cav3KO myoblast line in which an HA‐GFP‐tagged WT‐Cav3 was reintroduced. *Figure* 7A shows that we were able to detect an immunoreactive Cav3 band (~50 kDa) that corresponds to the HA‐GFP‐tagged WT‐Cav3, which was not seen in cell lysates from WT, Cav3KO, or Cav3KO cells expressing the empty vector (EV). The immunoreactive intensity of the 50 kDa band was similar to that of the native Cav3 band (at ~20 kDa) present in WT myoblasts suggesting Cav3 re‐expression in the null background was similar to that of WT cells. Immunoblotting cytosolic and mitochondrial fractions revealed that this re‐expressed HA‐GFP‐tagged Cav3 was detectable in the mitochondrial fraction (*Figure* 7B) and importantly restored expression of OPA1, MFN2, SDHA, ANT1, PGC1α, and MnSOD to levels that were comparable with that seen in WT‐L6 myoblasts (*Figure* 7C). As such, these changes were associated with a lowering of superoxide levels, restoration of mitochondrial membrane potential (*Figure* 7D), and improved respiratory function as assessed using Seahorse technology (*Figure* 7E).

### Caveolin‐3 deficiency modifies myocellular mitochondrial cholesterol and cardiolipin content

**Figure 8:**
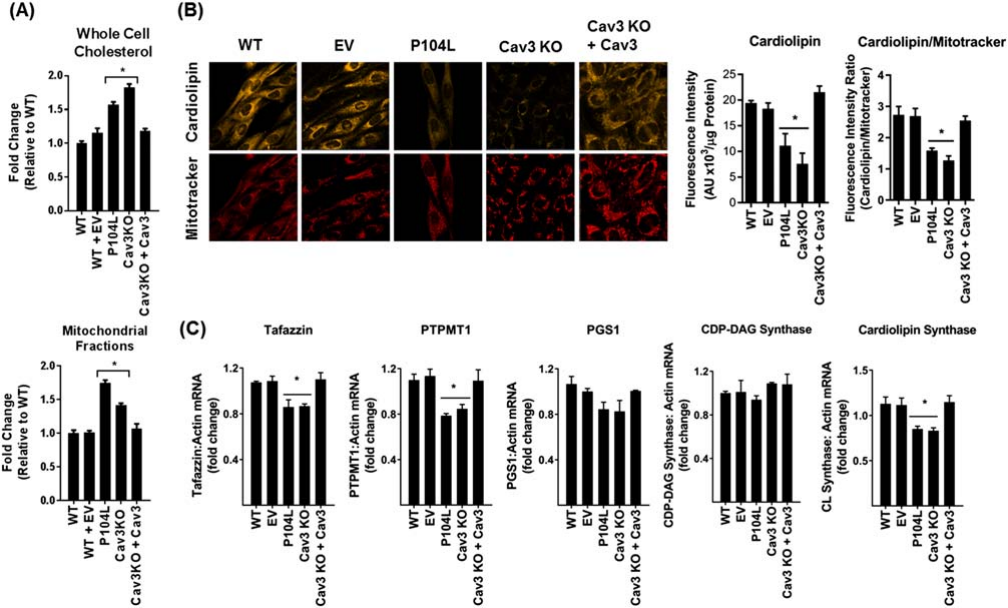
Effects of cellular Cav3 loss and Cav3 re‐expression on cholesterol and cardiolipin content in L6 muscle cells. Wild‐type (WT) L6 myoblasts or those transfected with an empty vector (EV) or stably expressing the Cav3^P104L^ LGMD1C mutation, or those in which Cav3 had been deleted by CRISPR/Cas9 (Cav3KO) and in which Cav3 was subsequently stably re‐expressed (Cav3KO + Cav3) were used to determine: (A) whole cell cholesterol (expressed relative to that assayed in WT cells) or cholesterol content of isolated mitochondrial membranes (expressed per unit protein content); (B) cardiolipin content using 10‐nonyl‐acridine orange (a cardiolipin probe) and mitotracker deep red prior to live cell confocal imaging (in which a minimum of 12 randomly chosen fields were chosen in three biological repeats) or quantification of fluorescence emission using a plate reader from three separate experiments each conducted in triplicate. The fluorescence intensity of cardiolipin in each cell condition was normalized to that of mitotracker for confocal‐based analysis or to protein content for plate reader‐based analysis; (C) expression of enzymes involved in cardiolipin biosynthesis in each cell condition was determined by quantitative polymerase chain reaction. All data are expressed as mean ± SEM from a minimum of three separate experiments. Asterisks indicate a significant change (*P* < 0.05) to the WT (control) value. PTPMT1, phosphatidylglycerophosphate and protein‐tyrosine phosphatase 1.

Given the important role that Cav plays in cellular cholesterol homeostasis, we postulated that Cav3 deficiency induced by expression of Cav3^P104L^ or by use of CRISPR/Cas9 gene editing may affect cholesterol content of muscle cells. In line with this, we observed a 57% and 82% increase in whole cell cholesterol in Cav3^P104L^ and Cav3KO myoblasts, respectively, (*Figure* 8A). Analysis of cholesterol content in isolated mitochondrial membrane fractions from these myoblasts revealed that it too was elevated in response to Cav3 deficiency. The increase in both whole cell and mitochondrial cholesterol was negated by re‐expression of Cav3 in Cav3KO myoblasts (*Figure* 8A).

Cardiolipin is a unique phospholipid located in the inner mitochondrial membrane, and its loss has been linked to mitochondrial dysfunction and a decline in muscle mass and strength.^48^ To assess whether cardiolipin content is sensitive to changes in Cav3 expression, we stained myoblasts with 10‐N‐nonyl acridine orange, a fluorescent cardiolipin indicator. Cardiolipin abundance was reduced significantly in both Cav3^P104L^ expressing myoblasts (by 43%) and Cav3KO muscle cells (by 62%) when quantified either per unit of cell protein or as a ratio to the fluorescence intensity of mitotracker (*Figure* 8B). This reduction in cardiolipin was mitigated upon stable re‐expression of Cav3 (*Figure* 8B). The presence of cardiolipin in the inner mitochondrial membrane is dependent upon a number of key biosynthetic enzymes such as tafazzin, phosphatidylgycerol phosphate synthase, phosphatidylglycerophosphate and protein–tyrosine phosphatase 1, cytidylyltransferase‐diacylglycerol synthase activity, and cardiolipin synthase. qPCR analysis assessing expression of genes encoding these enzymes indicated a significant decline in mRNA for tafazzin, PMTM1, and cardiolipin synthase in Cav3‐deficient myoblasts, whereas that for phosphatidylgycerol phosphate synthase and cytidylyltransferase‐diacylglycerol synthase was unaffected (*Figure* 8C).

## Discussion

Previous work has shown that the LGMD1C Cav3^P104L^ mutation is associated with aberrant accumulation of the mutant Cav3 protein within the Golgi where it forms unstable high molecular mass aggregates with wild type Cav3 and is subsequently targeted for degradation by the proteasome.^45^ A more recent study utilizing a transgenic Cav3 (p.P104L mutant) mouse model showed that such aggregation was a consequence of impaired ER‐Golgi protein processing, which, from proteomic profiling, was found to also affect proteins important for maintaining the integrity of the cytoskeleton, extracellular matrix, sarcolemma, and mitochondria.^11^ However, while this latter study detected changes in a total of 26 different mitochondrial proteins in muscle from p.P104L mice, no functional studies of mitochondrial respiration were performed. The work reported here demonstrates for the first time that (i) Cav3 appears to be closely associated with mitochondrial membranes prepared from mouse skeletal muscle and L6 myoblasts, (ii) loss of Cav3 in myoblasts expressing the P104L mutant or those in which the Cav3 gene has been silenced/deleted is associated with increased fragmentation of the mitochondrial network and a reduction in mitochondrial mass, (iii) that Cav3 loss induces deleterious changes in the expression of proteins regulating mitochondrial dynamics, redox status and respiratory function, and (iv) re‐expression of Cav3 in Cav3 deficient myoblasts not only promotes reformation of a tubular mitochondrial network but is also associated with improved mitochondrial redox status and respiratory function.

Although Cav1 immunoreactivity was detectable in crude homogenates prepared from mouse skeletal muscle, its abundance is substantially lower than that of Cav3, which is widely regarded as the principal Cav isoform in muscle^22^, ^49^ and, consequently, the one most likely to be of functional importance in this tissue. Consistent with this view, our data indicate that while Cav3^P104L^ induces a marked decline in total myocellular Cav3 and profound changes in mitochondrial morphology and function, it had little impact upon the abundance of Cav1. This observation implies that Cav1 is unlikely to substitute for Cav3 in skeletal muscle cells, at least in terms of helping to preserve mitochondrial integrity and function. This proposition is further supported by the fact that Cav1 was barely detectable in isolated mitochondrial fractions from mouse skeletal muscle and that myoblasts in which Cav1 has been deleted by CRISPR/Cas9 do not show any significant decline in cell respiration ( Supporting Information, *Figure* S5). However, it is noteworthy that while Cav1 appears dispensable for regulation of myocellular respiration, Cav1 loss in non‐muscle cell types (e.g. adipose tissue and mouse embryonic fibroblasts)^50^, ^51^, ^52^ has been linked to impaired mitochondrial function indicating that cell respiration and energy metabolism may be regulated in different tissues in a Cav isoform‐specific manner.

The reduced mitochondrial respiratory capacity associated with Cav3 deficiency is most likely the consequence of a number of factors, including a decline in mitochondrial mass that occurs in skeletal muscle of Cav3^*−/−*^ mice and myoblasts expressing Cav3^P104L^. This reduction in mitochondrial mass may be linked to changes in PGC1α, a key regulator of mitochondrial biogenesis, whose abundance was diminished in muscle of Cav3^*−/−*^ mice and L6 myoblasts in which Cav3 had been repressed by three different genetic approaches. PGC1α regulates the activity of several nuclear transcription factors, including the nuclear respiratory factors (NRF1 and NRF2) and the oestrogen‐related receptor α that, in turn, regulates expression of OXPHOS genes (e.g. cytochrome *c*, SDHA), mitochondrial uncoupling proteins (i.e. UCP3), ANT1, subunits of the mitochondrial ATP synthase, and ROS‐detoxifying enzymes.^47^, ^53^, ^54^ Consequently, the reduced transcriptional activation of genes encoding these functionally important mitochondrial proteins will impact negatively upon mitochondrial density and upon substrate (i.e. glucose) oxidation and ATP synthesis that is linked to electron transport chain (ETC) activity. In support of this proposition, we see a substantial decline (~70%) in the ATP:ADP ratio in Cav3KO myoblasts (Supporting Information, *Figure* S2E) and also find that while oxidation of glucose in WT myoblasts accounts for ~60% of ATP production, it only supports 20% of ATP‐linked respiration in Cav3KO myoblasts (Supporting Information, *Figure* S6A). It is possible that Cav3 deficiency may induce a metabolic shift towards greater anaerobic respiration, but analysis of the extracellular acidification rate, which is an indicative measure of lactate output and glycolytic flux, reveals that it too was significantly lower in Cav3KO myoblasts (Supporting Information, *Figure* S6B). These findings collectively signal that the decline in mitochondrial respiration in Cav3‐deficient myoblasts is associated with reduced demand for glucose as a metabolic fuel and is consistent with work showing that myoblasts expressing Cav3^P104L^ exhibit diminished glucose uptake.^18^

Modulation of mitochondrial dynamics may also influence respiratory function. Cav3 loss in myoblasts promotes a striking shift in mitochondrial morphology from a tubular network to one that was highly fragmented. While fission is physiologically important for maintaining mitochondrial quality control, excessive fragmentation of the mitochondrial network (due to increased fission and/or reduced mitochondrial fusion) results in accumulation of dysfunctional mitochondria, which, if functionally unrecoverable, are normally cleared by mitophagy. However, experiments assessing expression of a bifluorescent‐labelled (mCherry‐GFP) mitophagy reporter^55^, ^56^ in WT and Cav3KO myoblasts indicate that mitophagy was reduced (~20%) in Cav3KO myoblasts (Supporting Information, *Figure* S7). The reasons for this are currently unclear, but given that mitophagy represents selective degradation of mitochondria by autophagy and the latter process has significant energy and synthetic requirements, it is plausible that the energy deficit within Cav3KO cells compromises the effective clearance of damaged mitochondria.

Mitochondria are also the principal source of ROS (superoxide and hydrogen peroxide, H_2_O_2_) that are generated by reduction of oxygen by electrons ‘leaking’ from within the ETC during OXPHOS.^57^ In healthy cells, ROS production is normally low and rapidly neutralized by ROS detoxifying enzymes such as MnSOD. However, ROS generation can exceed the anti‐oxidant capacity of cells in numerous circumstances as seen, for example, during periods of excessive mitochondrial fuel supply and/or conditions that reduce expression/activity of anti‐oxidant enzymes.^56^ Our work indicates that muscle of Cav3^−/−^ mice and myoblasts expressing Cav3^P104L^ or those genetically depleted in Cav3 exhibit a significant reduction in MnSOD, which may contribute to the rise in mitochondrial superoxide. ETC components are very sensitive to reactive oxygen‐derived radicals, and their operational efficiency is not only reduced by oxidative stress but may further exacerbate ROS production that feeds a viscous cycle inducing ever greater mitochondrial dysfunction. This increased oxidative stress may also drive the observed changes in mitochondrial integrity and morphology. Previous work in C2C12 myoblasts has shown that acute exposure to H_2_O_2_ initiates mitochondrial fragmentation and that this is preceded by depolarisation of the mitochondrial membrane and a reduction in maximal respiration.^58^ Mitochondrial fragmentation is also a feature of muscle cells subjected to sustained fuel overloading.^56^ Interestingly, this fragmentation can be ameliorated by cell treatment with MitoTempo, a mitochondrial‐targeted superoxide scavenger. Notably, while mitochondrial fission is blunted by MitoTempo in fuel‐overloaded myotubes, it fails to mitigate the associated loss in mitochondrial respiratory capacity, which is likely sustained because of the failure to recover expression of key functional proteins, such as SDHA, ANT‐1, and UCP3.^56^ Based on these latter observations, the increase in oxidative stress seen in Cav3^P104L^ expressing myoblasts (and that reported in numerous muscular dystrophies^59^) may be important in influencing the morphology of the mitochondrial network. This possibility is supported by growing evidence linking changes in ROS to regulation of mitochondrial dynamics via non‐transcriptional modulation (e.g. phosphorylation, ubiquitination, and S‐nitrosylation) of mitochondrial fusion and fission proteins.^60^ Important in this context is the impact that an increase in superoxide in Cav3 depleted myoblasts may have upon proteins regulating mitochondrial dynamics. Myoblasts expressing Cav3^P104L^ (or those in which Cav3 was repressed by shRNA or CRISPR/Cas9) display a significant loss in both MFN2 and OPA1, whereas skeletal muscles of Cav3^−/−^ mice exhibit a two‐fold increase in Drp1. In either case, the observed changes would be consistent with a shift in mitochondrial dynamics towards greater fission. Whether this shift involves ROS‐mediated regulation of MFN2, OPA1, or Drp1 via changes in gene expression and/or post‐transcriptional modulation is currently unknown, but addressing this issue and how it might mechanistically link to changes in Cav3 expression represent important investigative goals of future work.

Although the cholesterol content of mitochondrial membranes is substantially lower than that of plasma membranes,^61^ it fulfils a vital role in their biogenesis, maintenance and membrane fluidity. Caveolins bind cholesterol with high affinity and play an important role in controlling cholesterol trafficking between multiple subcellular compartments, including mitochondria.^22^ Consequently, altered Cav expression/function may disturb intracellular trafficking/storage of cholesterol and partly be a cause for mitochondrial dysfunction. In line with this view, our results show that myoblasts expressing Cav3^P104L^ or those lacking Cav3 exhibit raised whole cell cholesterol that was also evident in mitochondrial membranes. While this increase may partly be linked to a loss in Cav‐dependent trafficking of the sterol through mitochondria, previous work by Bosch and coworkers has shown that mitochondrial cholesterol is also elevated in mouse embryonic fibroblasts lacking Cav1 despite there being no apparent localization of Cav1 in mitochondria in these cells.^51^ However, Cav1 does localize to specialized domains of the ER called mitochondrial associated membranes (MAMs) that potentially serve as foci for the movement of ions and lipids, including cholesterol, between the ER and mitochondria.^62^ Resident Cav1 would normally facilitate efflux of newly synthesized ER cholesterol to the plasma membrane, whereas Cav1 loss from MAMs would result in greater cholesterol ‘spillage’ to mitochondria. While we cannot fully exclude the possibility that the Cav3 we detect in our mitochondrial‐enriched fractions may also be of MAM origin, this seems unlikely given that, unlike VDAC or TOM20, these membrane fractions show no significant enrichment of BiP/GRP78, an ER marker protein. Regardless of whether Cavs are resident in mitochondria or MAMs, Bosch *et al*. showed that the increased mitochondrial cholesterol reduced mitochondrial membrane fluidity and, as shown in the current study, reduced respiratory activity and increased ROS production, which the authors attributed to a reduction in mitochondrial anti‐oxidant capacity.^51^ Significantly, the disturbances in cholesterol content, redox status, and mitochondrial function reported by Bosch *et al*. in Cav1‐deficient MEFs^51^ and by us in Cav3‐deficient myoblasts are recoverable upon cellular re‐expression of the respective Cav isoform. Furthermore, in our hands, this recovery not only involves restoration of the mitochondrial membrane potential but also reformation of the tubular mitochondrial network that is most likely facilitated by restraining loss of MFN2 and OPA1 that is otherwise seen in Cav3‐deficient myoblasts.

Another critical regulator of mitochondrial form and function is cardiolipin, a signature phospholipid found almost exclusively in the inner mitochondrial membrane where it plays a key role in many mitochondrial processes, including formation of cristae in the inner membrane, regulating the organization and activity of respiratory chain complexes and that of transporters (e.g. ANT‐1) and the ATP synthase.^63^, ^64^ A reduction in cardiolipin has been linked to mitochondrial dysfunction in numerous tissues, and our study reveals, for the first time, that this reduction is also a feature of myoblasts expressing the LGMD1C Cav3^P104L^ mutation and those in which the Cav3 gene has been deleted. Precisely how loss of Cav3 in myoblasts drives cardiolipin loss is unclear, but this decline may be linked to reduced expression of enzymes involved in its biosynthesis but also possibly its increased degradation. Cardiolipin is highly susceptible to ROS attack, and its peroxidation, as would be anticipated under conditions of heightened ROS generation, enhances its breakdown.^65^ Our work indicates that re‐expression of Cav3 not only mitigates the increase in ROS production but also normalizes expression of genes encoding cardiolipin synthase, protein–tyrosine phosphatase 1 and taffazin in Cav3KO myoblasts, which may collectively help restore cardiolipin content and improve mitochondrial integrity and function. Given the central role that cardiolipin plays in supporting a multitude of mitochondrial processes, there is considerable interest in exploiting this phospholipid as a drug target, especially in conditions where there is a deficit in mitochondrial energy production (e.g. skeletal muscle weakness, heart failure, and neurodegenerative disease). One potential therapeutic strategy involves use of Szeto‐Schiller peptides, which are small synthetic tetrapeptides that specifically target the activity of mitochondrial cytochrome *c* to inhibit cardiolipin peroxidation. These cardiolipin‐protective peptides have been shown to rejuvenate mitochondrial OXPHOS components, stimulate ATP production, and reduce electron ‘leakage’ from within the ETC and consequently ROS generation.^66^, ^67^ Whether cardiolipin loss serves as the primary trigger for the mitochondrial dysfunction we observe in myoblasts expressing Cav3^P104L^ is currently unknown, but assessing whether such peptide‐based interventions can attenuate the deleterious changes in mitochondrial morphology and function seen in these myoblasts is clearly worthy of further investigation.

In summary, our data reveal that Cav3 expression in skeletal muscle cells is an important regulator of mitochondrial homeostasis and that its loss, induced either by expression of the LGMD1C Cav3^P104L^ mutation or by targeted Cav3 gene silencing/deletion, reduces mitochondrial mass and promotes a profound morphological change in the mitochondrial network that impairs mitochondrial respiration and redox status. However, it is also important to stress that Cav3 loss in muscle cells induced by the above approaches has been shown to impair myoblast fusion and differentiation.^37^, ^38^, ^39^ Consequently, a potential limitation of the *in vitro* studies reported here is that because they were performed in myoblasts, this may have translational implications for understanding how muscle physiology is affected in patients presenting with caveolinopathies, such as LGMD1C. Whether Cav3 deficiency slows the process of myotube formation *in vivo* or modifies muscle fibre‐type composition is currently unknown, but it is noteworthy that studies utilizing a zebrafish model in which Cav3 was downregulated or the equivalent Cav3^P104L^ mutation was expressed display significant *in vivo* defects in myoblast fusion, muscle differentiation, and patterning during early development.^68^ Because myoblast fusion is an important determinant of muscle fibre size and Cav3 deficient mice have been shown to not only exhibit greater variability in fibre size but also increased fibre necrosis,^33^ it is plausible these myopathic changes are a consequence of maintaining the immature cell signature that has been identified *in vitro* in myoblasts expressing the P104L mutation.^37^ It is also conceivable that disturbances in mitochondrial biology and energy metabolism that we see associated with Cav3‐deficiency in myoblasts and mice contribute to the reduced fusion potential of myoblasts *in vitro* and to ultrastructural changes in muscle *in vivo*.^13^, ^34^ Whether skeletal muscle of LGMD1C patients also exhibit similar changes in mitochondrial biology is currently unknown, but testing this would be instructive not only for establishing the utility and relevance of myoblasts as a disease model but also for assessing whether impaired mitochondrial energetics/ATP production might contribute to muscle atrophy and the decline in muscle strength and exercise tolerance that has been reported in patients with caveolinopathies.^69^

## Conflict of interest

The authors confirm there are no conflicts of interests to declare.

## Supporting information


**Figure S1:** **Cav3 Co‐localises with mitochondria in L6 Muscle cells.**
Wild type (WT) L6 myoblasts stably transfected with WT‐Cav3‐GFP were stained with Mitotracker DeepRed and visualised using live cell confocal microscopy. Enlarged images (derived from the fields within the indicated white boxes) show co‐localisation (highlighted by white arrows) of WT‐Cav3‐GFP signal and Mitotracker Deep Red signal.The images are representative of two separate experiments.

**Figure S2:** **Cellular depletion of Cav3 induces changes in mitochondrial protein content, superoxide, membrane potential and ATP:ADP ratios.**
Whole cell lysates (WCL, 30 μg protein) from WT L6 myoblasts, short hairpin control (ShControl) stable transfected, or those in which Cav3 had been stably silenced using shRNA (shCav3) or deleted using CRISPR/Cas9 (Cav3KO) were subject to SDS‐PAGE and immunoblotted with antibodies to proteins shown (A) and their abundance quantified from a minimum of three separate experiments relative to actin (gel loading control) using Image J software (B). Alternatively, these cells were used for determination of superoxide content using fluorescence intensity (FI) of MitoSOX (C) and mitochondrial membrane potential using spectral analysis to monitor JC‐10 aggregate:monomer content from three separate experiments each conducted in triplicate. For these studies 5 μM FCCP was used as a positive control to help collapse the mitochondrial membrane potential (D) or for analysis of cellular ATP:ADP ratio (E). All graphical data represent mean ± SEM from three separate experiments. Asterisks indicate a significant change (*P* < 0.05), whereas the NS notation signifies no significant change. 
For analysis of ATP:ADP ratio muscle cells were grown to confluence in 6 cm culture dishes and prior to analysis of ATP and ADP washed with ice‐cold PBS. Cells were lysed in 5% (v/v) perchloric acid (PCA) and the samples were mixed to ensure complete lysis. Lysed cells were centrifuged at 18,000*g* for 3 min at 4°C and the supernatant used for further processing. PCA was neutralised with 2.5 M KOH in 1.1 M K_2_HPO_4_, after which the neutralised sample was mixed and centrifuged at 18,000*g* for 3 min. Adenine nucleotides within the supernatant were then separated by capillary electrophoresis with on‐column isotachophoretic concentration using buffers containing 50 mM sodium phosphate, 50 mM sodium chloride (pH 5.2; initial buffer) and 100 mM MES/Tris (pH 5.2; trailing buffer). To each buffer, 0.2% hydroxyethylcellulose was added to decrease electro osmotic flow. Nucleotide peaks were detected by UV absorbance at 260 nm and integrated using System Gold software. Peak areas, after correction of retention times, were used to calculate ratios. Retention times of ATP and ADP peaks were confirmed with samples spiked with internal standards (ATP and ADP) and analysis of the spectral absorbance of individual peaks.

**Figure S3:** **Effect of Cav3 loss on mitochondrial morphology in L6 myoblasts.**
WT L6 myoblasts or those transfected with a control shRNA and ShCav3 targeting and causing stable silencing of Cav3, or myoblasts subject to CRISPR/Cas9 to delete Cav3 (Cav3KO) were stained with Mitotracker Green prior to live cell confocal imaging to depict mitochondrial morphology. Enlarged images (derived from the fields within the indicated white boxes) highlight changes in mitochondrial morphology. Mitochondrial length was quantified using Volocity software and presented as elongated/tubular if greater than 1 μm and fragmented if less than 1μm in length. Data are presented as mean ± SEM from a minimum of three experiments in which at least 10 randomly chosen visual fields for each condition were analysed. Asterisks indicate a significant change (*P* < 0.05) between the black‐filled bars.

**Figure S4:** **The effect of myocellular Cav3 loss on mitochondrial respiration.**
WT L6 myoblasts or those transfected with a control shRNA or shRNA targeting and causing stable Cav3 loss (ShCav3) (A) or muscle cells subject to CRISPR/Cas9 to delete Cav3 (Cav3KO) (B) were subject to a ‘mitochondrial stress test’ in which the basal oxygen consumption rate (OCR) was measured using Seahorse technology. Oligomycin (1 μM), FCCP (1 μM) and a mixture of Rotenone (1 μM)/Antimycin (2 μM) were added at the times indicated by dotted lines to help infer of basal, ATP‐linked and maximal respiration. The Seahorse traces shown in A and B are from a single experiment in which each point represents the mean ± SEM from triplicate analyses. The bar graph data represents the analysis of three individual experiments (values are mean ± SEM). Asterisks indicate significant change (*P* < 0.05) between bars specified.

**Figure S5:** **Effects of Cav1 deficiency on mitochondrial respiration in L6 myoblasts.**
Wild type (WT) L6 myoblasts and myoblasts in which Cav1 had been deleted by CRISPR/Cas9 (Cav1 KO) (Sense gRNA: AGTGTACGACGCGCACACCAAGG Antisense gRNA: GGTACCGTCTGCTCCACTTACTC) were subject to a ‘mitochondrial stress test’ using a Seahorse XF24 analyser to allow determination of basal oxygen consumption, ATP‐linked respiration and maximal respiration. All data are presented as mean ± SEM from three experiments. NS indicates no significant change**.**

**Figure S6:** **Defining the contribution of glucose use for ATP‐linked respiration and analysis of extracellular acidification rates (ECAR) in wild type and Cav3‐deficient L6 myoblastst.**
Wild type L6 myoblasts, control short hairpin transfected (shControl) myoblasts or those in which Cav3 had been stably silenced by shRNA (shRNACav3) or deleted by CRISPR/Cas9 (Cav3KO) were used as shown in the two panels to measure (A) the component of ATP‐linked respiration that was dependent on glucose oxidation or (B) determine extracellular acidification rates (ECAR) as a measure of anaerobic glycolytic flux using the seahorse XF24 analyser. For the experiment in (A), WT and Cav3KO myoblasts were incubated in the absence and presence of 20 mM 2‐deoxyglucose (2DG, a glycolytic inhibitor) to assess the effect of blocking glucose use on the oligomycin‐sensitive (i.e. ATP‐linked) respiration. The experiments in (A) represent data from 5 experimental determinations whereas analysis of ECAR (B) was based on three experimental determinations. All data are presented as mean ± SEM. Asterisks indicate significant change (*P* < 0.05) between bars specified while NS indicates no significant change.

**Figure S7:** **Effects of Cav3 depletion on mitophagy in L6 myoblasts.**
For analysis of mitophagy we infected myoblasts with a retroviral construct encoding a tandem mCherry‐GFP tag attached to the outer mitochondrial membrane localization signal of Fis1 (residues 101–152). In myoblasts expressing this construct, mitochondria will fluoresce red and green. However, upon increased mitophagy, mitochondria are delivered to lysosomes where the low pH quenches the GFP signal but not mCherry. Consequently, the degree of mitophagy in wild type (WT) and Cav3‐deficient myoblasts can be calculated by assessing the relative change in their red:green signal ratio using fluorescence‐activated cell sorting (FACS), a specialized form of flow cytometry using the indicated gating strategy (A). WT and Cav3 knockout (Cav3KO) cell lines infected with the retrovirus containing a tandem mCherry‐GFP protein (selected for using hygromycin) were utilised for flow cytometry after which the geometric mean intensity of both mCherry and GFP signalling was determined and used to calculate the ratio of mCherry to GFP in both cell lines as an indicator of mitophagy (B). Data are presented as mean ± SD from two experiments each with two replicates of 20,000 cells**.**
