# Supplementary figures and images for: Caveolin‐3 deficiency associated with the dystrophy P104L mutation impairs skeletal muscle mitochondrial form and function
Source: J Cachexia Sarcopenia Muscle. 2020 Feb 23;11(3):838–58. doi: 10.1002/jcsm.12541 (PMC7296273; doi:10.1002/jcsm.12541)

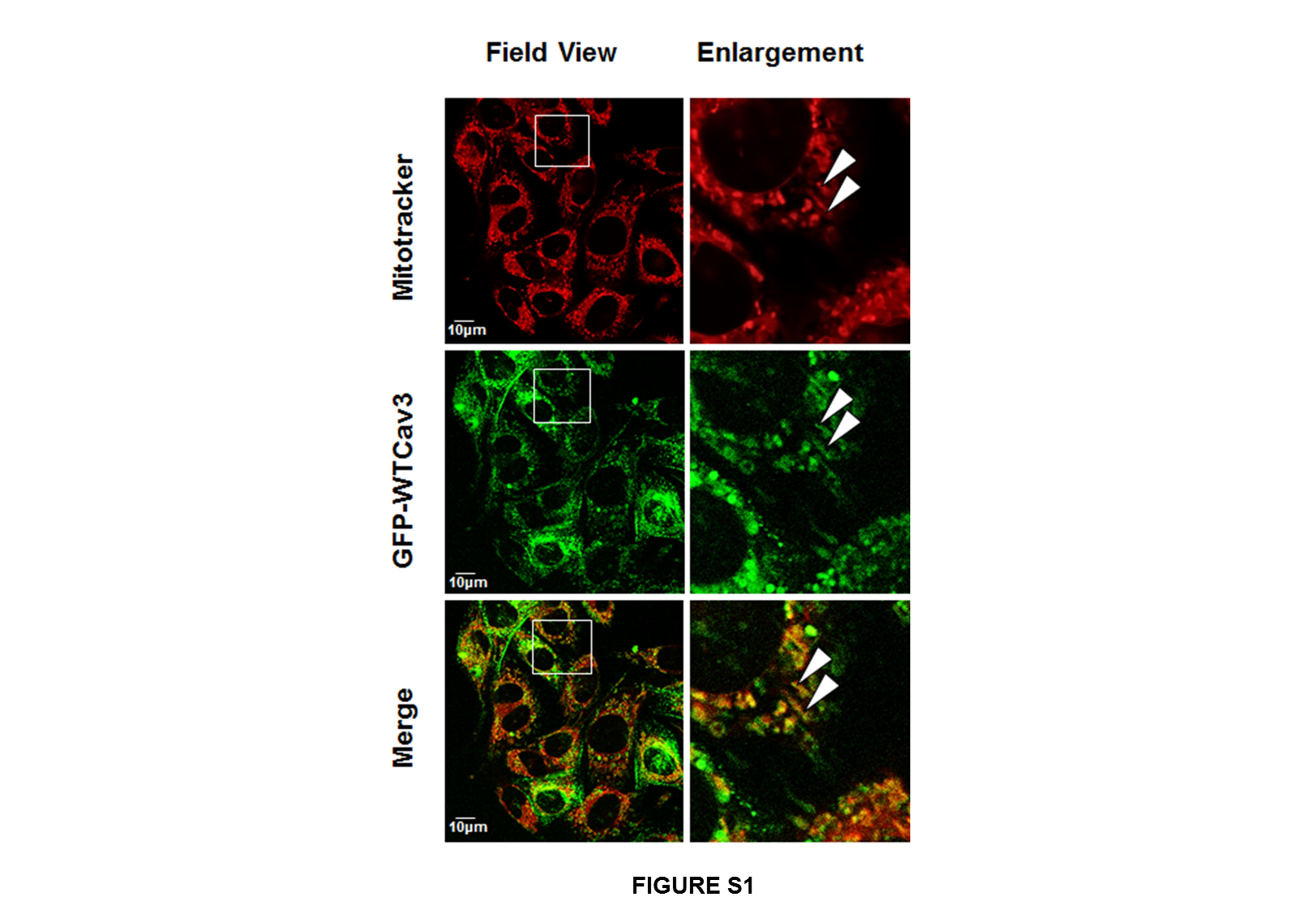

Supplement: Supplementary file 1 — Figure S1: Cav3 Co‐localises with mitochondria in L6 Muscle cells. Wild type (WT) L6 myoblasts stably transfected with WT‐Cav3‐GFP were stained with Mitotracker DeepRed and visualised using live cell confocal microscopy. Enlarged images (derived from the fields within the indicated white boxes) show co‐localisation (highlighted by white arrows) of WT‐Cav3‐GFP signal and Mitotracker Deep Red signal.The images are representative of two separate experiments. [file JCSM-11-838-s001.tif]

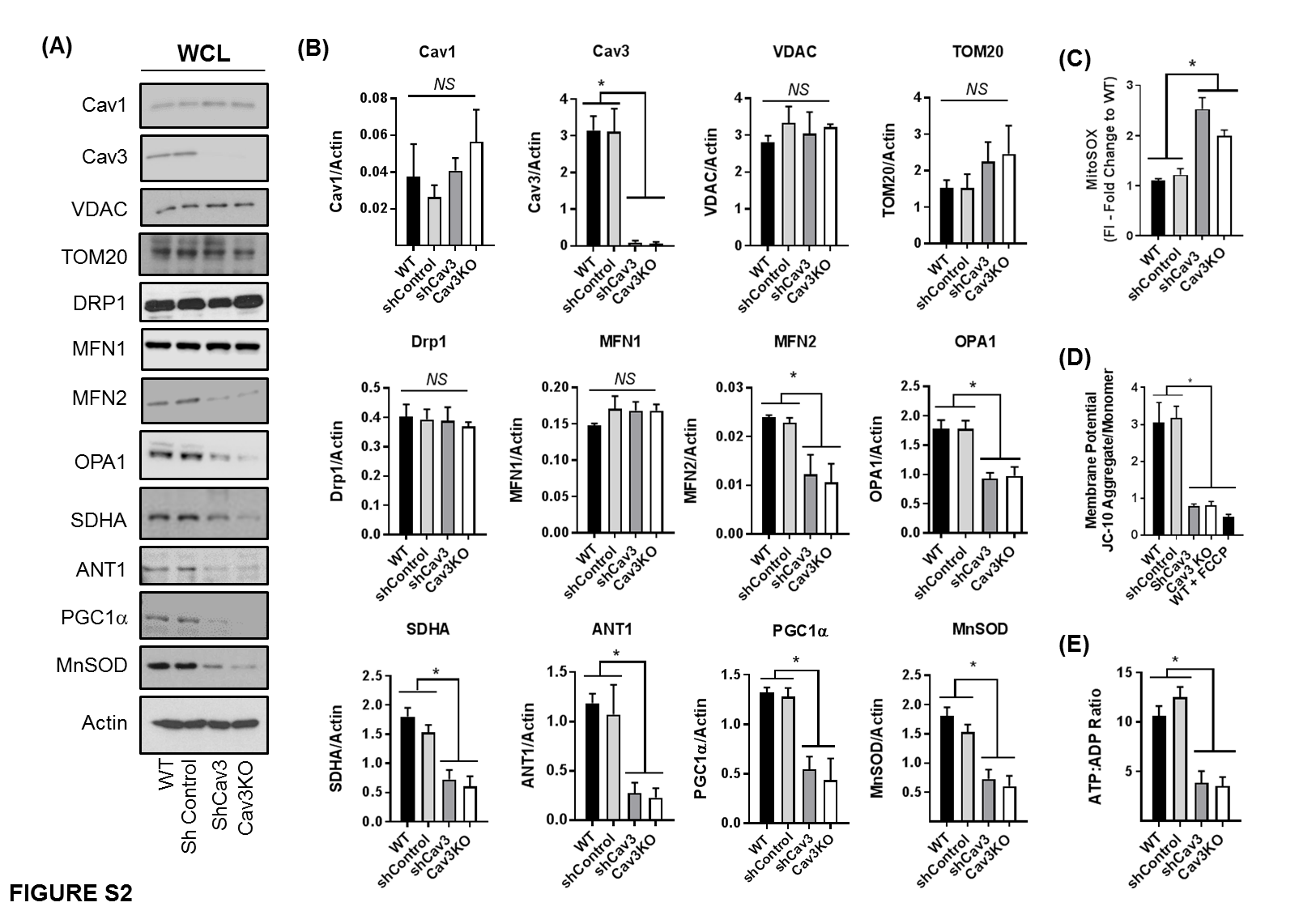

Supplement: Supplementary file 2 — Figure S2: Cellular depletion of Cav3 induces changes in mitochondrial protein content, superoxide, membrane potential and ATP:ADP ratios. Whole cell lysates (WCL, 30 μg protein) from WT L6 myoblasts, short hairpin control (ShControl) stable transfected, or those in which Cav3 had been stably silenced using shRNA (shCav3) or deleted using CRISPR/Cas9 (Cav3KO) were subject to SDS‐PAGE and immunoblotted with antibodies to proteins shown (A) and their abundance quantified from a minimum of three separate experiments relative to actin (gel loading control) using Image J software (B). Alternatively, these cells were used for determination of superoxide content using fluorescence intensity (FI) of MitoSOX (C) and mitochondrial membrane potential using spectral analysis to monitor JC‐10 aggregate:monomer content from three separate experiments each conducted in triplicate. For these studies 5 μM FCCP was used as a positive control to help collapse the mitochondrial membrane potential (D) or for analysis of cellular ATP:ADP ratio (E). All graphical data represent mean ± SEM from three separate experiments. Asterisks indicate a significant change (P < 0.05), whereas the NS notation signifies no significant change. For analysis of ATP:ADP ratio muscle cells were grown to confluence in 6 cm culture dishes and prior to analysis of ATP and ADP washed with ice‐cold PBS. Cells were lysed in 5% (v/v) perchloric acid (PCA) and the samples were mixed to ensure complete lysis. Lysed cells were centrifuged at 18,000g for 3 min at 4°C and the supernatant used for further processing. PCA was neutralised with 2.5 M KOH in 1.1 M K2HPO4, after which the neutralised sample was mixed and centrifuged at 18,000g for 3 min. Adenine nucleotides within the supernatant were then separated by capillary electrophoresis with on‐column isotachophoretic concentration using buffers containing 50 mM sodium phosphate, 50 mM sodium chloride (pH 5.2; initial buffer) and 100 mM MES/Tris (pH 5.2; trailing buff [file JCSM-11-838-s002.tif]

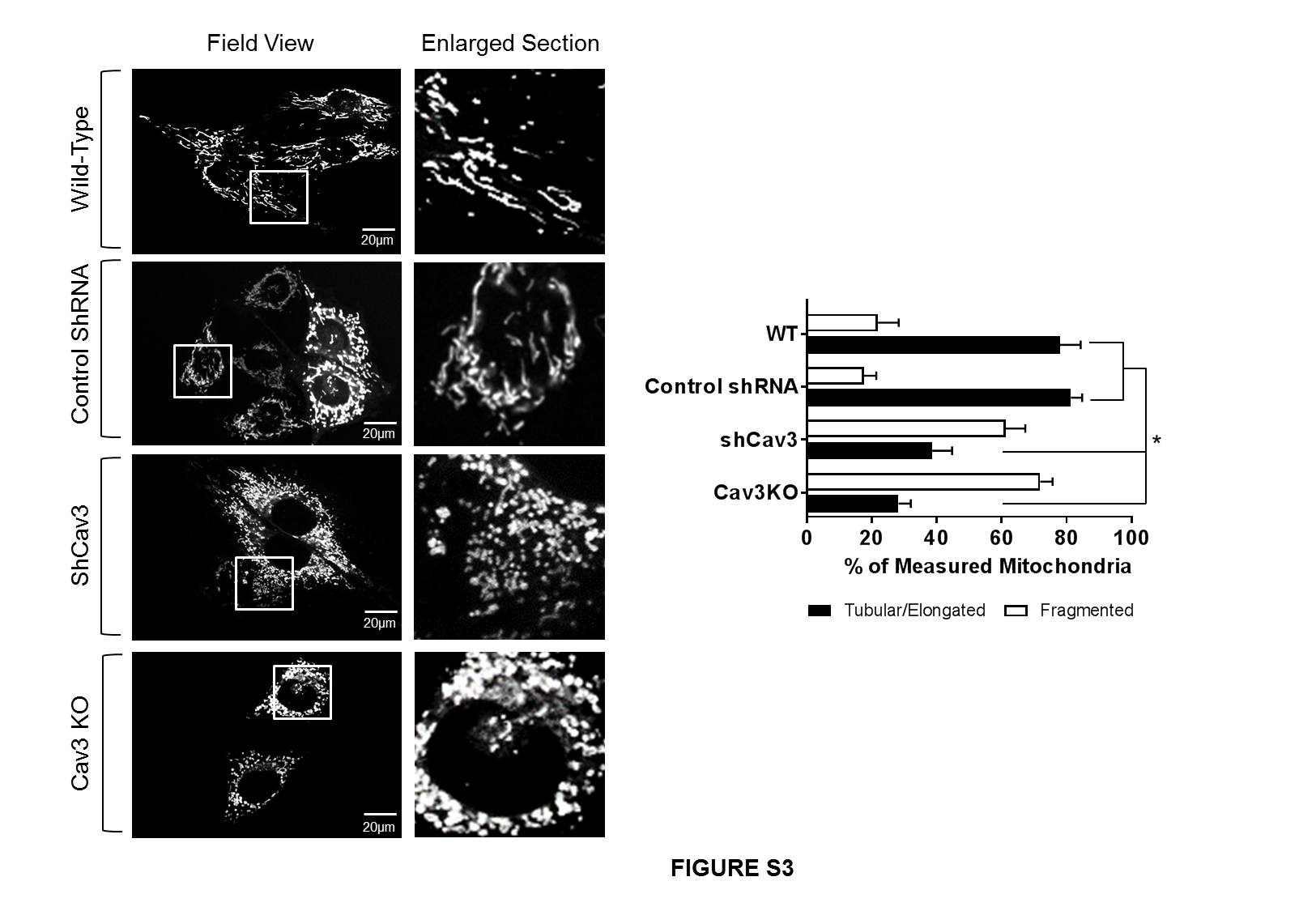

Supplement: Supplementary file 3 — Figure S3: Effect of Cav3 loss on mitochondrial morphology in L6 myoblasts. WT L6 myoblasts or those transfected with a control shRNA and ShCav3 targeting and causing stable silencing of Cav3, or myoblasts subject to CRISPR/Cas9 to delete Cav3 (Cav3KO) were stained with Mitotracker Green prior to live cell confocal imaging to depict mitochondrial morphology. Enlarged images (derived from the fields within the indicated white boxes) highlight changes in mitochondrial morphology. Mitochondrial length was quantified using Volocity software and presented as elongated/tubular if greater than 1 μm and fragmented if less than 1μm in length. Data are presented as mean ± SEM from a minimum of three experiments in which at least 10 randomly chosen visual fields for each condition were analysed. Asterisks indicate a significant change (P < 0.05) between the black‐filled bars. [file JCSM-11-838-s003.tif]

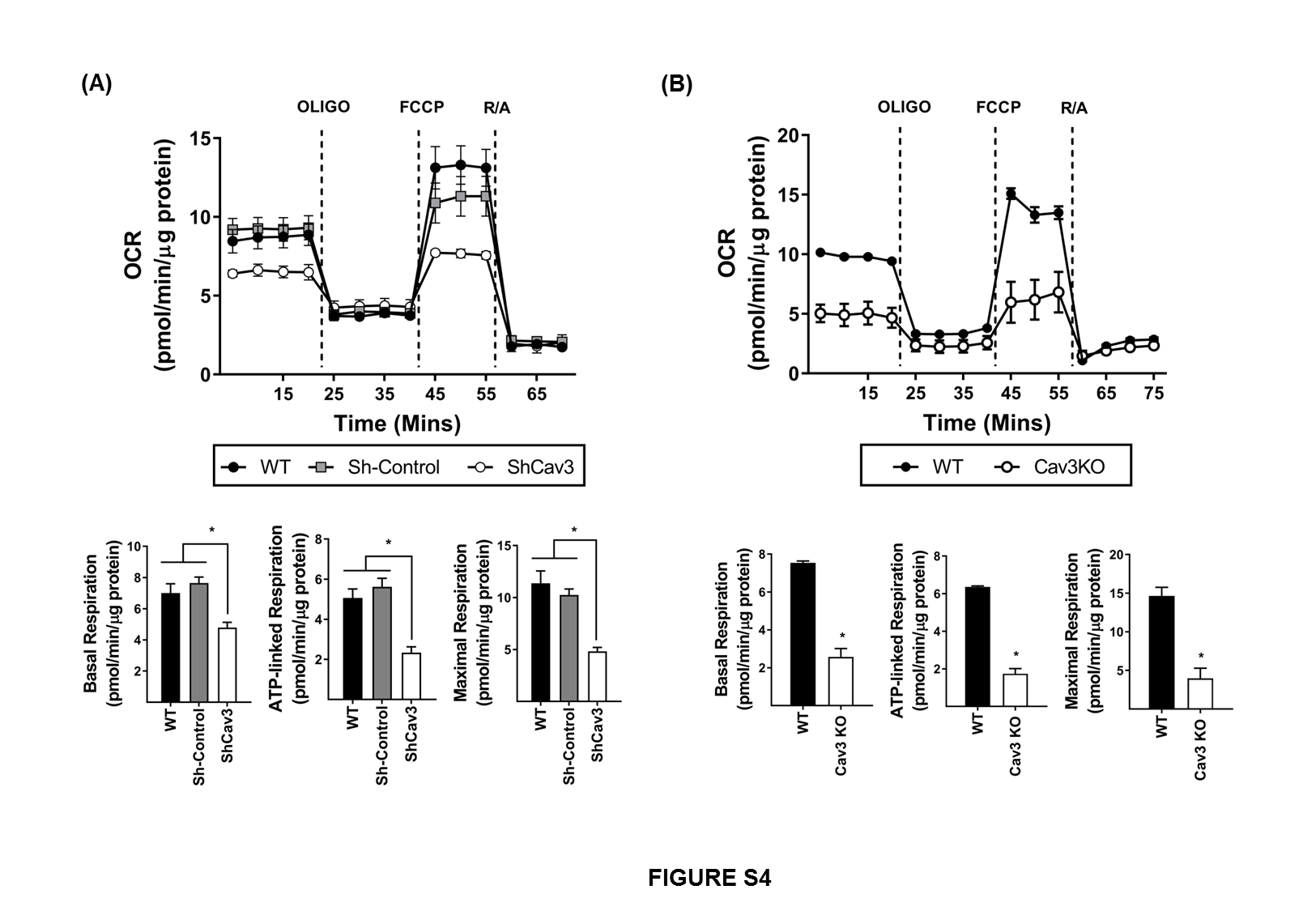

Supplement: Supplementary file 4 — Figure S4: The effect of myocellular Cav3 loss on mitochondrial respiration. WT L6 myoblasts or those transfected with a control shRNA or shRNA targeting and causing stable Cav3 loss (ShCav3) (A) or muscle cells subject to CRISPR/Cas9 to delete Cav3 (Cav3KO) (B) were subject to a ‘mitochondrial stress test’ in which the basal oxygen consumption rate (OCR) was measured using Seahorse technology. Oligomycin (1 μM), FCCP (1 μM) and a mixture of Rotenone (1 μM)/Antimycin (2 μM) were added at the times indicated by dotted lines to help infer of basal, ATP‐linked and maximal respiration. The Seahorse traces shown in A and B are from a single experiment in which each point represents the mean ± SEM from triplicate analyses. The bar graph data represents the analysis of three individual experiments (values are mean ± SEM). Asterisks indicate significant change (P < 0.05) between bars specified. [file JCSM-11-838-s004.tif]

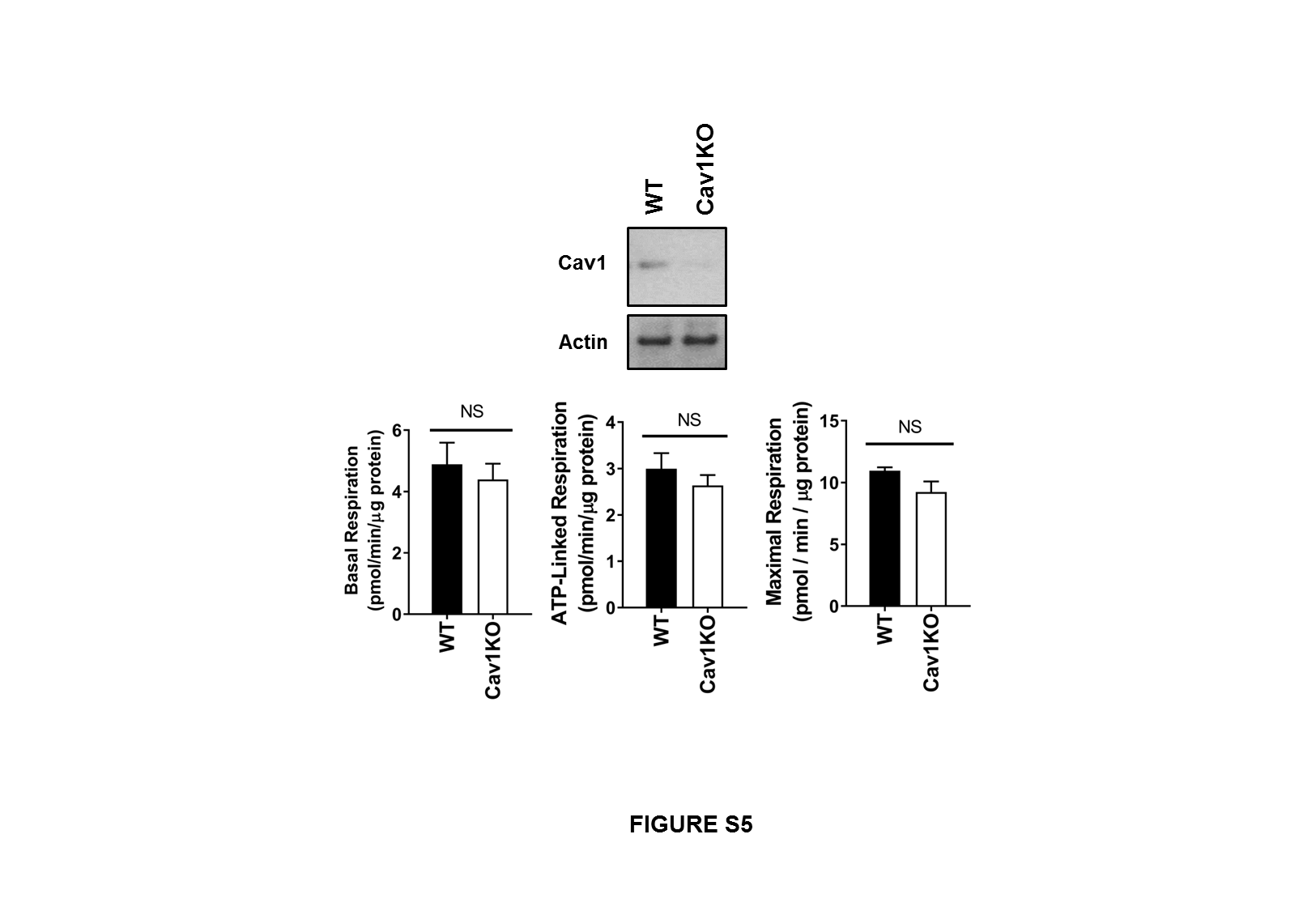

Supplement: Supplementary file 5 — Figure S5: Effects of Cav1 deficiency on mitochondrial respiration in L6 myoblasts. Wild type (WT) L6 myoblasts and myoblasts in which Cav1 had been deleted by CRISPR/Cas9 (Cav1 KO) (Sense gRNA: AGTGTACGACGCGCACACCAAGG Antisense gRNA: GGTACCGTCTGCTCCACTTACTC) were subject to a ‘mitochondrial stress test’ using a Seahorse XF24 analyser to allow determination of basal oxygen consumption, ATP‐linked respiration and maximal respiration. All data are presented as mean ± SEM from three experiments. NS indicates no significant change. [file JCSM-11-838-s005.tif]

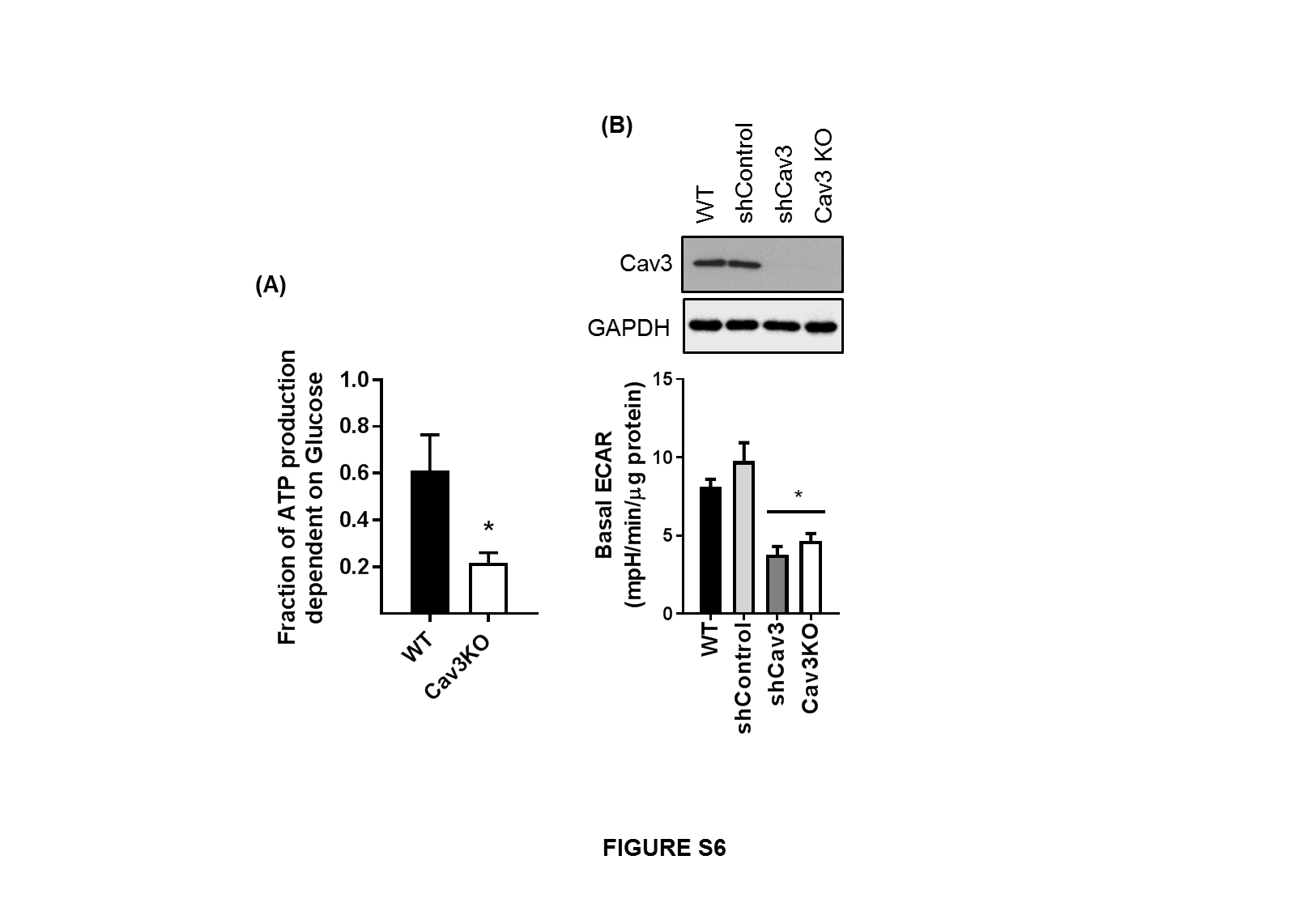

Supplement: Supplementary file 6 — Figure S6: Defining the contribution of glucose use for ATP‐linked respiration and analysis of extracellular acidification rates (ECAR) in wild type and Cav3‐deficient L6 myoblastst. Wild type L6 myoblasts, control short hairpin transfected (shControl) myoblasts or those in which Cav3 had been stably silenced by shRNA (shRNACav3) or deleted by CRISPR/Cas9 (Cav3KO) were used as shown in the two panels to measure (A) the component of ATP‐linked respiration that was dependent on glucose oxidation or (B) determine extracellular acidification rates (ECAR) as a measure of anaerobic glycolytic flux using the seahorse XF24 analyser. For the experiment in (A), WT and Cav3KO myoblasts were incubated in the absence and presence of 20 mM 2‐deoxyglucose (2DG, a glycolytic inhibitor) to assess the effect of blocking glucose use on the oligomycin‐sensitive (i.e. ATP‐linked) respiration. The experiments in (A) represent data from 5 experimental determinations whereas analysis of ECAR (B) was based on three experimental determinations. All data are presented as mean ± SEM. Asterisks indicate significant change (P < 0.05) between bars specified while NS indicates no significant change. [file JCSM-11-838-s006.tif]

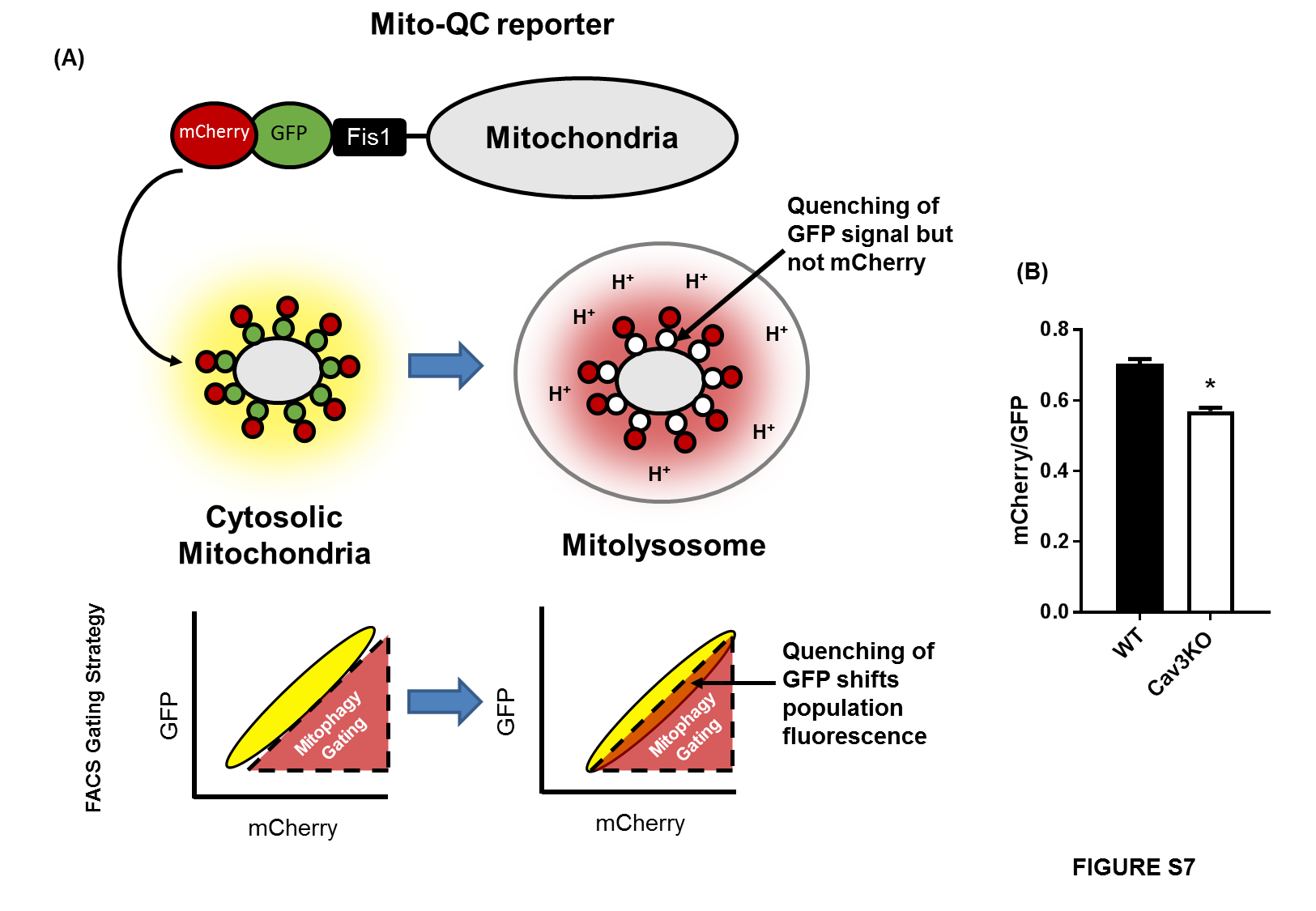

Supplement: Supplementary file 7 — Figure S7: Effects of Cav3 depletion on mitophagy in L6 myoblasts. For analysis of mitophagy we infected myoblasts with a retroviral construct encoding a tandem mCherry‐GFP tag attached to the outer mitochondrial membrane localization signal of Fis1 (residues 101–152). In myoblasts expressing this construct, mitochondria will fluoresce red and green. However, upon increased mitophagy, mitochondria are delivered to lysosomes where the low pH quenches the GFP signal but not mCherry. Consequently, the degree of mitophagy in wild type (WT) and Cav3‐deficient myoblasts can be calculated by assessing the relative change in their red:green signal ratio using fluorescence‐activated cell sorting (FACS), a specialized form of flow cytometry using the indicated gating strategy (A). WT and Cav3 knockout (Cav3KO) cell lines infected with the retrovirus containing a tandem mCherry‐GFP protein (selected for using hygromycin) were utilised for flow cytometry after which the geometric mean intensity of both mCherry and GFP signalling was determined and used to calculate the ratio of mCherry to GFP in both cell lines as an indicator of mitophagy (B). Data are presented as mean ± SD from two experiments each with two replicates of 20,000 cells. [file JCSM-11-838-s007.tif]
